# Phenotypic diversity in an endangered freshwater fish *Squalius microlepis* (Actinopterygii, Leuciscidae)

**DOI:** 10.3897/zookeys.897.38768

**Published:** 2019-12-09

**Authors:** Nina G. Bogutskaya, Oleg A. Diripasko, Primož Zupančič, Dušan Jelić, Alexander M. Naseka

**Affiliations:** 1 Naturhistorisches Museum Wien, Burgring 7, Vienna 1010, Austria Naturhistorisches Museum Wien Vienna Austria; 2 Croatian Institute of Fisheries and Marine Ecology, 8 Konsulska St, Berdyansk, 71118, Ukraine Croatian Institute of Fisheries and Marine Ecology Berdyansk Ukraine; 3 Dolsko 14, 1262 Slovenia Unaffiliated Dolsko Slovenia; 4 Institute for Biodiversity, Croatian Biological Research Society, 7 Lipovac I, 10000, Zagreb, Croatia Institute for Biodiversity, Croatian Biological Research Society Zagreb Croatia

**Keywords:** Biodiversity, freshwater fishes, variability and polymorphism, distribution, Dinaric karst

## Abstract

*Squalius microlepis* was examined from recent and historical collections within the known range of the species with special emphasis on intraspecific variability and variations, and compared to its closest relative species *S. tenellus* (in total, 193 specimens; 33 absolute and 52 proportional measurements and ratios, and 12 counts including vertebrae). *Squalius tenellus* was perfectly differentiated in all statistical analyses and can be diagnosed by 76–95 (vs. 64–80) scales in lateral series, 68–83 (vs. 58–77) lateral-line scales, (17)18–20 (vs. 13–16(17)) scales above lateral line, and (7)8–10 (vs. 4–7) scales below lateral line. *Squalius microlepis* was morphologically heterogeneous, with two phenotypes readily distinguishable (phenotype 1 corresponding to *S. microlepis* s. str. as defined by its lectotype) by a combination of many characters; those contributing most to the discrimination were number of gill rakers, length of lower jaw (% interorbital width), and head length (% SL). Only phenotype 1 was found in the Ričina-Prološko Blato-Vrljika karst system; most of the specimens from the lower Matica and the Tihaljina-Trebižat karst system were identified as phenotype 2; the sample from karstic poljes near Vrgorac contained both phenotype 1 and 2, and individuals of intermediate morphology. As very limited molecular data exist on the two phenotypes of *S. microlepis*, we refrain from any taxonomic conclusions until new molecular approaches (and new markers) are used. We also report on a dramatic reduction of the area of distribution and abundance of *S. microlepis* in recent years.

## Introduction

The genus *Squalius* Banaparte is widely distributed throughout Europe and the Middle East, and shows an especially high diversity in the Mediterranean basin. Approximately 50 species are currently recognised in the genus (Kottelat and Freyhof 2007; Turan et al. 2009; Bogutskaya and Zupančič 2010; Zupančič et al. 2010; Özuluğ and Freyhof 2011), and sixteen species are known to occur in Europe (Özuluğ and Freyhof 2011).

Small-scaled chubs, *S. microlepis* Heckel, 1843 and *S. tenellus* Heckel, 1843, are superficially similar but distinguishable based on scale counts according to Bănărescu and Herzig-Straschil (1998): 67–75 vs. 76–85 total lateral line scales, 24–26 vs. 28–32 circumpeduncular scales, 13–15 vs. 15–17 scales in a transverse row between the dorsal-fin origin and the lateral line, 5–6 vs. 6–7 scales in a transverse row between the lateral line and the pelvic-fin origin in *S. microlepis* vs. *S. tenellus.*

Available data on genetic markers for *Squalius microlepis* and *S. tenellus* show that they form a sister-pair in a clade, which is restricted to the Iberian and Apennine Peninsulas and the eastern Adriatic basin (Perea et al. 2010; Geiger et al. 2014; Schönhuth et al. 2018). However, the genetic markers differ in their resolution of phylogenetic relationships between the two species. The CO1 mitochondrial marker do not distinguish them (Perea et al. 2010; Geiger et al. 2014) while mitochondrial cytb, a combined nuclear data set (RAG+S7), and the combined mitochondrial and nuclear data sets CO1+cytb+RAG+S7 (Perea et al. 2010) and CO1+cytb+RAG+S7 (Schönhuth et al. 2018) support some divergence.

*Squalius tenellus* is distributed in karstic waters of Livanjsko Polje including Buško Blato (Buško Jezero), an accumulation lake, located in the southern part of Livanjsko Polje and northwest of Duvajnsko Polje; Mandečko Lakes and in Blidinje Lake to where it was supposedly introduced over 100 years ago (Bănărescu and Herzig-Straschil 1998; Kottelat and Freyhof 2007; Zupančič 2008). PZ found this species in a stream at Glamoč in Glamočko Polje located in the northeast of Livanjsko Polje and west of Kupreško Polje. Data on distribution presented by Crivelli (2006) and Freyhof and Kottelat (2008) on sympatric distribution of *S. microlepis* and *S. tenellus* in lakes Buško and Mandečko near Livno may probably reflect different taxonomic opinions of the authors on synonymisation of the two species. Ćurčić (1915) reported *S. tenellus* from Mostarsko Blato (repeated by Karaman (1928: 160)) that have been confirmed by recent studies (Šanda et al. 2008, 2010). *Squalius tenellus* was allegedly introduced into the Cetina River drainage and this river is included in the range of this species by some authors (Freyhof and Kottelat 2008; Ćaleta et al. 2015).

Recent summarising publications (Habeković and Pažur 1995; Bănărescu and Herzig-Straschil 1998; Bogut et al. 2006; Mrakovčić et al. 2006, 2016; Kottelat and Freyhof 2007; Zupančič 2008; Šanda et al. 2009; Ćaleta et al. 2015, 2019) indicate that the range of *S. microlepis* encompasses the entire karst system of the Culuša – Ričina – Brina – Suvaja – Matica – Vrljika – Tihaljina – Mlade – Trebižat (a single river interrupted by underground sections, a tributary to the Neretva) downstream to the waterfall Kravice. In this karst river system, it occurs in basins of the Prološko Blato Lake and the Ričice Reservoir in the Imotski region in Croatia and in Krenica Lake and the Matica, Vrljika, Tihaljina and Trebižat rivers in Bosnia and Herzegovina. It was found outside the Matica-Vrljika-Tihaljina-Trebižat system further southwards in the Neretva drainage – in the Matica River at Imotski in Polje Jezero [Vrgoraska Matica River, do not be confused with Matica-Vrljika] and reported from Baćina lakes of the lower Neretva. The species is known under a vernacular name ‘masnica’ or ‘mašnica’ in western Herzegovina (Bosnia and Herzegovina) and ‘makal’ (‘makali’ or ‘makalj’) in Croatia including in the Vrgorac area (Heckel and Kner 1858: 206, Ćaleta et al. 2019: 168).

An examination of *S. microlepis* samples, deposited in the historical fish collection at Museum of Natural History in Vienna and recent collections, revealed some morphological heterogeneity of the species. The goal of this study was a comparative morphological analysis of the group of the small-scaled Adriatic *Squalius* (*S. tenellus* and *S. microlepis*) to approach issues of its morphological diversity. The study on intraspecific morphological differences was aimed at contributing, in the future, to integrative phylogenetic analyses and species delimitations in the group.

*Squalius microlepis* was assessed by IUCN at global level as endangered (EN B2ab(ii, iii)) ver. 3.1 (Crivelli 2006), and in Croatian national Red book it was assessed as critically endangered (CR A1ace, C2a(iii)) (Mrakovčić et al. 2006). It is strictly protected by Nature protection Acts in both Croatia and Bosnia and Herzegovina.

## Materials and methods

**Figure 1.**
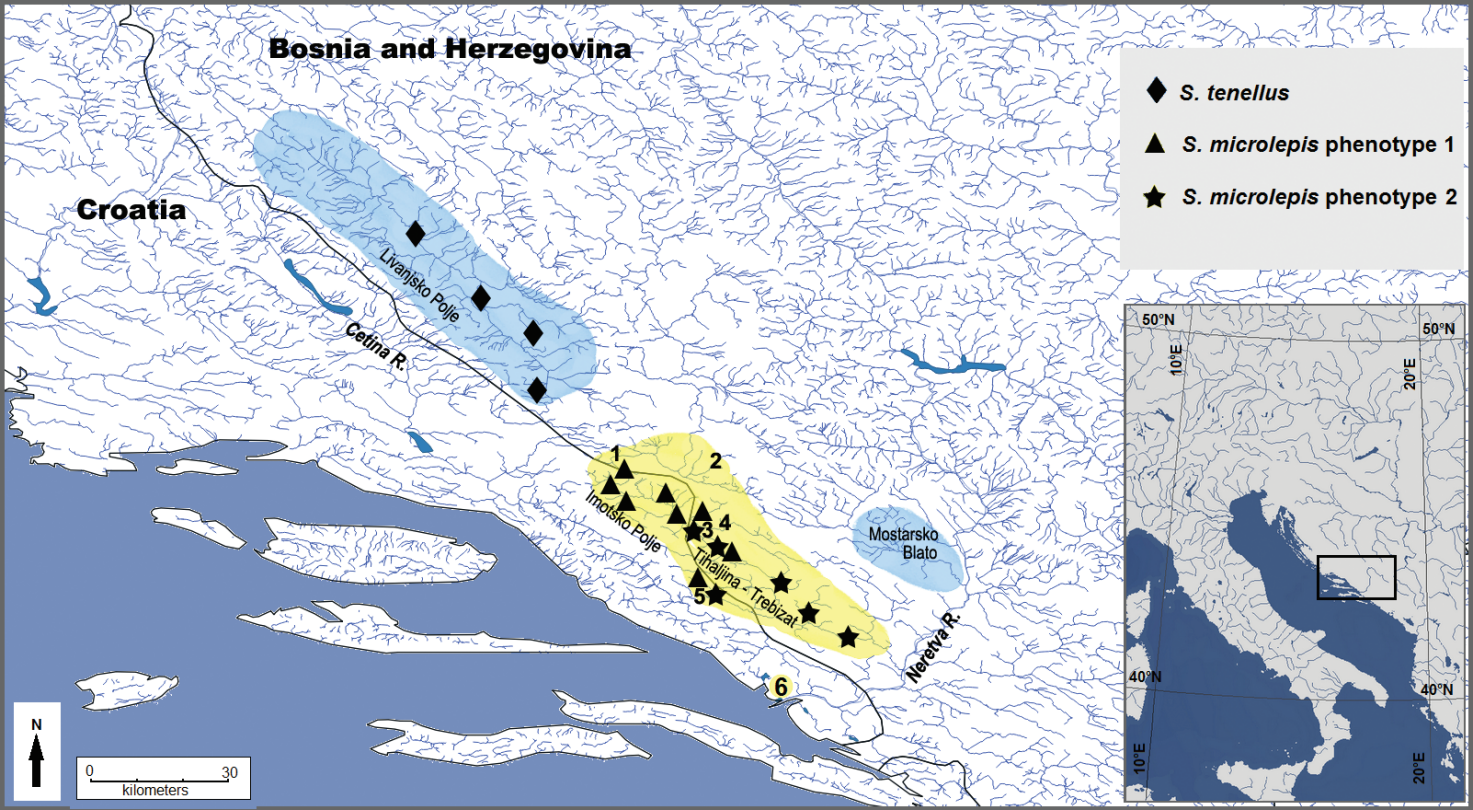
Map showing localities of examined specimens: *S. tenellus*, *S. microlepis* phenotype 1, and *S. microlepis* phenotype 2, shadowed areas showing ranges of *S. tenellus* (blue) and *S. microlepis* (yellow); 1 – Ričice Reservoir, 2 – Ričina River, Posušje, 3 – Lower Matica River, 4 – Krenica Lake, 5 – Vrgoračka Matica River system (Vrgoračko Polje, Polje Jezero), 6 – Baćina lakes.

In total, 193 specimens were examined, material see Table 1; examined localities are presented in Fig. 1. Most examined specimens were available in collections. Those specimens collected in the wild using SAMUS 725MP (Samus Special Electronics, Poland) (max. 1000V, 650W) electrofishing device and hand nets were euthanised with etheric clove oil (*Eugenia caryophyllata*) diluted in water (5 drops of oil per 5 l of water) and preserved in 5% formaldehyde and then stored in 70% ethanol.

**Table 1.** Examined material.

Area	Sample data	Identification (present study)
**Ričina-Prološko Blato-Vrljika, Krenica Lake**	**Imotsko Polje (Croatia)**	*Squalius microlepis* phenotype 1
	NMW 49413, 2, 84.8–98.2 mm SL, ‘Imosky’, 1886, no collector;	
	NMW 49415, lectotype, 151.2 mm SL, ‘Imosky, Kroatien (Dalmatien), Heckel Reise 1840’;	
	NMW 49414, 3 paralectotypes, 75.4–108.6 mm SL, data as lectotype;	
	NMW 49416, 1 paralectotype, 139.6 mm SL, data as lectotype;	
	NMW 49421, 1 paralectotype [not 3 as given by Bănărescu and Herzig-Straschil (1998: 417)], 149.9 mm SL, data as lectotype;	
	NMW 49417, 3, 95.5–98.1 mm SL, Imosky 1886, no collector;	
	NMW 49418, 2, 86.5–92.8 mm SL, same as 49417;	
	NMW 49419, 2, 86.5 mm SL, same as 49417;	
	NMW 49420, 2, 102.3–107.7 mm SL, same as 49417;	
	NMW 49422, 1, ‘Prolozac bei Imotski’, 1904, Kolombatowitsch;	
	MNCN_ICTIO 291.725–291.729, 4, 147.5–166.8 mm SL, Prološko Blato [Proložac] Lake, 8 May 2008;	
	PZC 283, 3, 160.5–186.2 mm SL, same locality and collector as above, 2 July 2004;	
	PZC 545, 5, 145.2–206.5 mm SL, same locality and collector as above, 16 Aug. 2008.	
	**Vrljika River (Croatia)**	*Squalius microlepis* phenotype 1
	NMW 12729-732, 4, 119.5–121.7 mm SL, ‘Vrlica-Fluss bei Imotski’, no date, no collector;	
	NMW 49399, 4, 118.6–149.4 mm SL, Vrlica, Imotski, 1901, coll. Sturany;	
	NMW 49400, 3, 113.3–121.2 mm SL, same data;	
	NMW 49401, 2, 153.2–155.6 mm SL, same data;	
	NMW 49402, 3, 138.4–145.5 mm SL, same data;	
	NMW 49403, 3, 132.2–161 mm SL, same data;	
	NMW 49404, 2, 180.7–215 mm SL, same data;	
	NMW 49405, 3, 142.6–158.1 mm SL, same data;	
	NMW 49406, 3, 133.5–217 mm SL, same data;	
	NMW 49407, 3, 163.7–192.5 mm SL, same data;	
	NMW 49408, 3, 102.6–106.4 mm SL, same data;	
	NMW 49409, 2, 156.6–158.7 mm SL, same data;	
	NMW 49410, 2, 135.3–137.1 mm SL, same data;	
	NMW 49411, 2, 136.6–152.5 mm SL, same data;	
	NMW 49412, 2, 143.3–190.5 mm SL, same data;	
	NMW 49221, 2, 191.2–203.9 mm SL, same data.	
	**Ričina River (Croatia)**	*Squalius microlepis* phenotype 1
	MNCN_ICTIO 294.784–294.800, 17, 70.9–223.3 mm SL, Ričice Reservoir (Ričina River), coll. Zupančič, 22 Apr. 2004;	
	MNCN_ICTIO 292.541–292.545, 5, 165.7–223.4 mm SL, same locality and collector as above, 16 Aug. 2008.	
	PZC 501, 16, 53.2–135.6 mm SL, same locality and collector as above, 1 May 1999.	
	**Krenica Lake (Bosnia and Herzegovina)**	*Squalius microlepis* phenotype 1
	MNCN_ICTIO 295.855–295.860, 6, 61.1–116.1 mm SL, Krenica Lake at Drinovci, 43°22'26"N, 17°19'56"E, coll. Zupančič, 17 July 2002;	
	MNCN_ICTIO 296.096–296.097, 2, 71.6, 147.6 mm SL, same locality and collector as above, 7 July 2011.	
**Lower Matica-Tihaljina-Trebižat**	**Lower Matica River (Bosnia and Herzegovina)**	*Squalius microlepis* phenotype 2
	MNCN_ICTIO 292.120–292.123, 2, 174.9, 177.8 mm SL, Matica River at Drinovci, 43°21'29"N, 17°17'29"E, coll. Zupančič, 4 Aug. 2007;	
	ZISP 54994, 5, 96.3–147.2 mm SL, same locality as above, coll. Zupančič, 7 July 2011.	
	**Tihaljina River (Bosnia and Herzegovina)**	*Squalius microlepis* phenotype 2
	All from Tihaljina River at bridge in Tihaljina, 43°18'27"N, 17°23'22"E; coll. Zupančič:	
	NMW 95294, 3, 98.6–173.9 mm SL, 4–5 Aug. 2007;	
	MNCN_ICTIO 294.588–294.594, 7, 72.0–194.4 mm SL, 15 Aug. 2001;	
	MNCN_ICTIO 294.548–294.552, 5, 104.2–192.2 mm SL, 16 Aug. 2001;	
	MNCN_ICTIO 293.145–293.147, 3, 126.1–156.3 mm SL, 2 June 2008;	
	MNCN_ICTIO 294.596–294.599, 4, 108.2–158.3 mm SL, 9 July 2008;	
	MNCN_ICTIO 292.129–292.136, 5, 96.4–222.9 mm SL, 4 Oct. 2009;	
**Lower Matica-Tihaljina-Trebižat**	PZC 531, 1, 255.7 mm SL, 4–5 Aug. 2007;	*Squalius microlepis* phenotype 2
	uncat., 3, 94.3–153.5 mm SL, 3 June 2000.	
	**J**: out of MNCN_ICTIO 292.129–292.136, 127.05 mm SL, Tihaljina River at bridge in Tihaljina, 43°18'27"N, 17°23'22"E; coll. Zupančič, 4 Oct. 2009.	*Squalius microlepis* phenotype 1
	**K**: 149.75 mm SL, as J.	
	**Trebižat River: (Bosnia and Herzegovina)**	*Squalius microlepis* phenotype 2
	MNCN_ICTIO 294.472–294.473, 2, 140.1, 152.6 mm SL, Trebižat River at bridge between Grabovnik and Vašarovići, 43°12'38"N, 17°29'03"E, coll. Zupančič, 8 July 2011.	
**Presumably, from polijes at Vrgorac**	**A**: NMW 49428, 1, 165.8 mm SL, ‘Lago di Dusino presso Imosky’, 1848, coll. Parreyss.	*Squalius microlepis*, intermediate between phenotypes
**Neretva drainage, uncertain**	**B**: NMW 49427, 1, 140.1 SL mm, ‘Narenta, Heckel Reise 1840’.	*Squalius microlepis*, intermediate between phenotypes
**Vrgoracko Polje and Polje Jezero karst system (Croatia)**	**C**: NMW 49424, 1, 168.1 mm SL, ‘Vergoraz [See Jessero], Heckel Reise 1840’.	*Squalius microlepis* phenotype 1
	**D**: NMW 49425, 1, 178.0 mm SL, ‘See zw. Gradač and Vrgorač’, 1888, don. Scharfetter.	*Squalius microlepis*, intermediate between phenotypes
	**E**: NMW 49426, 1, 193.8 mm SL, ‘See zw. Gradač and Vrgorač’, 1888, don. Scharfetter.	*Squalius microlepis* phenotype 1
	**F**: NMW 49423:1, 122.5 mm SL, ‘Vergoraz [See Jessero], Heckel Reise 1840’.	*Squalius microlepis* phenotype 2
	**G**: NMW 49423:2, 276.1 mm SL, as F.	*Squalius microlepis* phenotype 2
**Presumable not Zadar but Neretva drainage, uncertain**	**H**: NMW 49228:1, 165.8 mm SL, Zara [Zadar](see text for discussion on locality), no date, coll. Kolombatović.	*Squalius microlepis* phenotype 1
	**I**: NMW 49228:2, 205.1 SL, as H.	
	NMW 16001, lectotype, 122.1 mm SL, Livno [Livanjsko Polje], [Heckels Reise, 1840]; NMW 16002, 2 paratypes, 78.6 mm and 73.9 mm SL, data as lectotype;	*Squalius tenellus*
	NMW 49613, 2 paratypes, 94.9 mm and 82.7 mm SL, data as lectotype;	
	MNCN_ICTIO 292.166–292.168, 3, 137.5–183.9 mm SL, stream at Glamoč [Glamočko Polje], ca. 44°1'56"N 16°53'44"E, coll. Zupančič, 17 Aug. 2009;	
	MNCN_ICTIO 293.014–293.016, 4, Žabljak R. at Žabljak, north from Livno [Livanjsko Polje], 43°48'45"N 16°59'51"E, coll. Zupančič, 13 Aug. 2001.	
**Buško Reservoir**	MNCN_ICTIO 294.142–294.158, 17, 165.0–205.4 mm SL, Buško Blato at Prisoje, ca. 43°40'54"N 17°4'14"E, coll. Zupančič, 22 Apr. 2004.	*Squalius tenellus*

The fin insertion is the posterior-most point where the last fin ray connects with the body. Measurements follow Kottelat and Freyhof (2007) except that head length (HL), eye diameter, postorbital length and interorbital width include the skin fold. All measurements were made point-to-point with an electronic calliper and recorded to the nearest of 0.1 mm. Standard length was measured from the anteriormost extremity of the upper lip to the posterior margin of the hypurals at midline. Maximum body depth was measured at the deepest section of the body which is about the middle of distance between the nape and the dorsal-fin origin. Body depth was also measured in front of the dorsal-fin origin. Additional measurements of the cranium, jaws and operculum were made point to point from the anteriormost extremity to the posteriormost extremity (lengths), from the uppermost extremity to the lowermost extremity (depths), and between the lateralmost extremities (widths). Length of the cranial roof was measured from the anterior margin of the supraethmoid to the base of the supraoccipital crest. Characters include 33 absolute and 52 proportional measurements and ratios and 12 counts as given in Tables 2–5. Vertebral counts and terminology follow Naseka (1996). A qualitative character “a point where the dorso-hypural distance, which is taken from the dorsal-fin origin to the end of the hypural complex, falls when reported forward” follows Doadrio et al. (2007) and Kottelat and Freyhof (2007: fig. 2). The last two branched rays articulating on a single pterygiophore in the dorsal and anal fins are noted as “1½”. Total number of scales in the lateral series (bearing the lateral-line canal or without the canal; equal number of transverse rows of scales) included scales at the caudal-fin base. Total number of lateral-line (pored / bearing the lateral-line canal) scales included scales at the caudal-fin base. Scale counts in a transverse row above and below the lateral line (in transverse row between dorsal-fin origin and lateral line, and in transverse row between lateral line and pelvic-fin origin, respectively) follow Kottelat and Freyhof (2007: fig. 10). Gill rakers count included all gill rakers on both lower and upper limb of the arch. Fin-ray counts and axial skeleton characters were examined from radiographs.

**Table 2.** Morphometric and meristic data of *Squalius microlepis* phenotypes 1 and 2 and *Squalius tenellus* identified based on preliminary examination (see text for explanations).

	***S. microlepis* phenotype 1, *N* = 47**				***S. microlepis* phenotype 2, *N* = 46**				***S. tenellus*, *N* = 25**			
	**min**	**max**	**mean**	**sd**	**min**	**max**	**mean**	**sd**	**min**	**max**	**mean**	**sd**
SL, mm	61.14	223.44	**142.16**	46.47	72.04	255.72	**146.10**	36.67	122.10	205.39	**171.56**	21.49
Maximum body depth (% SL)	21.05	26.71	**23.67**	1.37	20.91	25.06	**22.87**	1.04	22.03	26.58	**24.38**	1.09
Depth of caudal peduncle (% SL)	9.06	10.61	**9.85**	0.44	9.47	11.01	**10.25**	0.42	9.34	11.30	**10.50**	0.42
Depth of caudal peduncle (% length of caudal peduncle)	45.72	56.58	**51.06**	3.02	45.92	59.48	**51.88**	3.34	49.29	59.24	**53.52**	2.72
Body width at dorsal-fin origin (% SL)	10.54	16.04	**13.47**	1.02	13.09	17.71	**15.06**	1.09	12.91	18.30	**14.48**	1.36
Caudal peduncle width (% SL)	6.80	10.26	**8.41**	0.76	7.75	11.08	**9.37**	0.62	7.77	10.70	**8.98**	0.80
Predorsal length (% SL)	56.11	61.20	**58.49**	1.14	54.33	58.54	**56.46**	1.14	54.96	58.56	**56.75**	0.93
Postdorsal length (% SL)	30.29	35.77	**32.27**	1.28	31.93	35.81	**34.38**	0.83	31.94	36.99	**33.89**	1.20
Prepelvic length (% SL)	50.90	59.63	**55.25**	2.28	49.54	54.27	**51.86**	1.09	53.00	57.03	**54.84**	1.14
Preanal length (% SL)	69.93	78.71	**73.67**	2.21	70.02	74.39	**72.07**	1.13	71.32	77.38	**73.86**	1.18
Pectoral – pelvic-fin origin length (% SL)	22.86	29.33	**26.08**	1.45	22.79	29.14	**26.03**	1.30	25.54	29.44	**27.79**	1.04
Pelvic – anal-fin origin length (% SL)	17.40	23.61	**19.63**	1.18	18.96	23.16	**20.98**	0.98	18.32	21.79	**20.06**	0.85
Length of caudal peduncle (% SL)	16.88	21.77	**19.34**	1.04	17.07	21.62	**19.81**	0.95	17.44	21.68	**19.65**	1.15
Dorsal-fin base length (% SL)	9.38	13.28	**11.09**	0.77	9.23	13.57	**11.37**	0.91	10.73	12.33	**11.66**	0.44
Dorsal fin depth (% SL)	13.94	18.89	**16.19**	1.11	14.32	18.86	**15.94**	1.14	11.42	18.20	**16.06**	1.28
Anal-fin base length (% SL)	8.07	12.38	**10.07**	0.96	9.62	12.34	**10.70**	0.59	9.37	16.07	**10.64**	1.22
Anal fin depth (% SL)	10.21	15.76	**12.42**	0.96	10.42	14.45	**11.99**	0.83	10.14	13.84	**12.17**	0.81
Pectoral fin length (% SL)	15.36	19.09	**17.64**	0.90	14.68	19.97	**17.15**	0.99	15.33	19.26	**17.17**	0.96
Pelvic fin length (% SL)	12.96	15.89	**14.07**	0.61	12.56	15.53	**13.93**	0.74	11.94	15.17	**13.95**	0.77
Head length (% SL)	28.97	33.67	**31.06**	1.34	25.39	29.62	**27.38**	0.95	26.87	29.97	**28.91**	0.77
Head length (% body depth)	113.99	148.29	**131.59**	8.51	108.52	138.39	**119.94**	6.37	104.48	129.69	**118.79**	5.89
Head depth at nape (% SL)	16.34	20.35	**17.92**	0.84	15.84	18.39	**16.99**	0.56	16.60	18.86	**17.69**	0.62
Head depth at nape (% HL)	52.35	61.23	**57.74**	2.21	58.18	66.80	**62.10**	2.29	56.15	65.36	**61.22**	2.35
Head depth through eye (% HL)	36.43	46.06	**41.04**	2.06	40.16	47.39	**43.35**	1.76	38.58	47.87	**43.32**	2.66
Maximum head width (% SL)	13.07	15.90	**14.43**	0.58	13.08	15.56	**14.18**	0.57	12.39	16.68	**14.75**	0.94
Maximum head width (% HL)	40.97	52.29	**46.54**	2.54	45.81	57.88	**51.85**	2.67	42.33	57.60	**51.05**	3.42
Snout length (% SL)	7.90	10.65	**9.18**	0.57	7.75	9.23	**8.41**	0.34	7.70	9.38	**8.75**	0.38
Snout length (% HL)	26.97	32.35	**29.57**	1.52	27.35	33.57	**30.75**	1.48	28.31	32.78	**30.26**	1.05
Eye horizontal diameter (% SL)	4.56	7.94	**5.95**	1.04	4.12	6.62	**5.07**	0.64	4.33	5.98	**4.81**	0.34
Eye horizontal diameter (% HL)	14.09	25.74	**19.13**	3.14	14.96	23.38	**18.51**	2.03	14.94	21.99	**16.66**	1.51
Eye horizontal diameter (% interorbital width)	46.77	86.54	**64.30**	11.65	44.02	69.51	**55.27**	7.27	44.19	69.52	**50.30**	5.02
Postorbital distance (% HL)	49.98	58.10	**54.13**	2.07	51.83	57.43	**54.39**	1.37	53.92	58.84	**56.56**	1.20
Interorbital width (% SL)	8.04	10.25	**9.27**	0.49	8.53	9.80	**9.19**	0.33	8.60	10.46	**9.59**	0.44
Interorbital width (% HL)	26.71	32.92	**29.89**	1.54	30.44	36.57	**33.61**	1.43	30.45	36.58	**33.16**	1.43
Length of upper jaw (% HL)	27.04	34.12	**30.32**	1.59	27.21	33.03	**29.41**	1.01	29.04	33.73	**30.37**	1.09
Length of upper jaw (% SL)	7.92	11.35	**9.42**	0.61	7.42	8.89	**8.05**	0.33	8.01	9.41	**8.78**	0.33
Length of lower jaw (% SL)	11.28	13.70	**12.46**	0.64	9.25	11.08	**10.23**	0.42	10.00	12.04	**11.09**	0.44
Length of lower jaw (% HL)	35.94	43.94	**40.13**	1.65	34.80	40.52	**37.37**	1.05	34.72	40.26	**38.37**	1.37
Length of lower jaw (% interorbital width)	121.44	154.57	**134.50**	6.51	99.74	120.89	**111.35**	4.58	101.98	128.28	**115.92**	6.76
Length of lower jaw (% depth of operculum)	107.34	128.60	**117.57**	4.98	89.15	114.07	**102.71**	4.63	100.00	129.14	**111.63**	7.76
Cranium width between margins of pterotics (% cranium roof length)	60.08	76.79	**68.11**	3.23	58.95	81.63	**71.15**	4.38	64.53	75.25	**69.14**	3.38
Cranium width between margins of sphenotics (% cranium roof length)	49.71	64.45	**56.58**	3.46	51.25	68.40	**61.08**	3.47	54.87	64.87	**59.49**	2.81
Cranium width between margins of supraethmoid (% cranium roof length)	19.74	26.51	**23.63**	1.75	20.05	26.51	**23.65**	1.42	19.38	28.32	**23.66**	2.17
Cranium width between margins of supraethmoid (% cranium width between margins of pterotics)	28.21	40.53	**34.77**	3.04	28.87	37.41	**33.28**	1.74	30.04	41.08	**34.22**	2.68
Length of lower jaw (% cranium width between margins of pterotics)	95.09	118.46	**105.16**	5.91	81.34	100.86	**89.81**	4.10	88.51	109.80	**98.87**	6.45
Depth of operculum (% HL)	31.35	38.65	**34.18**	1.65	32.11	41.52	**36.45**	1.81	29.91	38.86	**34.48**	1.93
RATIOS:												
Interorbital width/eye horizontal diameter	1.16	2.14	**1.61**	0.28	1.44	2.27	**1.84**	0.24	1.44	2.26	**2.00**	0.17
Snout length/eye horizontal diameter	1.06	2.24	**1.59**	0.31	1.17	2.13	**1.68**	0.22	1.29	2.10	**1.83**	0.17
Head depth at nape/eye horizontal diameter	2.16	4.29	**3.10**	0.55	2.56	4.23	**3.40**	0.42	2.97	4.23	**3.70**	0.29
Head length/caudal peduncle depth	2.77	3.48	**3.16**	0.21	2.35	3.02	**2.68**	0.16	2.41	2.95	**2.76**	0.13
Length of caudal peduncle/caudal peduncle depth	1.77	2.19	**1.97**	0.12	1.68	2.18	**1.94**	0.12	1.69	2.03	**1.87**	0.09
Length of lower jaw/caudal peduncle depth	1.06	1.43	**1.27**	0.09	0.88	1.15	**1.00**	0.06	0.92	1.15	**1.06**	0.06
Pectoral fin length/pectoral – pelvic-fin origin distance	0.60	0.83	**0.68**	0.06	0.56	0.77	**0.66**	0.05	0.53	0.69	**0.62**	0.04
Predorsal length/head length	1.75	2.00	**1.89**	0.07	1.93	2.20	**2.06**	0.07	1.83	2.08	**1.96**	0.06
COUNTS:												
Scales in lateral series	67	78	**72.33**	2.83	64	77	**69.77**	3.22	76	95	**85.48**	4.98
Lateral-line scales	58	77	**68.47**	3.91	58	75	**67.70**	3.99	68	83	**76.72**	4.34
Scales above lateral line	13	16	**14.49**	0.74	13	16	**14.25**	0.78	17	20	**18.80**	0.82
Scales below lateral line	5	7	**6.16**	0.69	4	7	**5.61**	0.72	7	10	**8.76**	0.78
Gill rakers	14	16	**15.21**	0.67	11	14	**12.59**	0.84	14	18	**15.72**	0.98
Number of predorsal vertebrae	15	16	**15.19**	0.39	14	16	**14.86**	0.41	15	16	**15.16**	0.37
Number of abdominal vertebrae	24	25	**24.67**	0.47	24	25	**24.55**	0.50	24	25	**24.40**	0.50
Number of caudal vertebrae	17	19	**17.79**	0.67	17	20	**18.50**	0.59	17	20	**18.64**	0.76
Total vertebrae	42	44	**42.47**	0.67	42	44	**43.05**	0.43	42	44	**43.04**	0.54
Difference between abdominal and caudal numbers	5	8	**6.88**	0.96	4	8	**6.05**	1.01	4	8	**5.76**	1.16

**Table 3.** Frequency of occurrence of diagnostic meristic character states in *Squalius microlepis* phenotypes 1 and 2 and in *S. tenellus.*

	**Number of scales in lateral series**								**Number of scales above lateral line**								**Number of scales below lateral line**							**Gill rakers**							
	**64–67**	**68–71**	**72–75**	**76–79**	**80–83**	**84–87**	**88–91**	**92–95**	**13**	**14**	**15**	**16**	**17**	**18**	**19**	**20**	**4**	**5**	**6**	**7**	**8**	**9**	**10**	**11**	**12**	**13**	**14**	**15**	**16**	**17**	**18**
*S. tenellus*, *N* = 28				**6**	**5**	**9**	**5**	**3**					**1**	**9**	**13**	**5**				**1**	**9**	**14**	**4**				**1**	**12**	**10**	**3**	**2**
*S. microlepis* phenotype 1																															
Ričice Reservoir, *N* = 38	4	12	17	5					1	16	18	2	1					5	23	10							7	15	16		
Prološko Lake, Imotski *N* = 30	3	12	13	2					2	12	14	2						7	14	9							8	14	8		
Vrljika, *N* = 43	3	18	16	4	2				4	18	14	4						8	17	15						1	11	23	9		
Krenica Lake, *N* = 8		5	3						2	5	1							5	2	1							2	5	1		
Total, *N* = 119	**10**	**48**	**49**	**11**	**2**				**9**	**53**	**48**	**9**	**1**					**26**	**58**	**36**						**1**	**28**	**57**	**34**		
*S. microlepis* phenotype 2																															
Tihaljina and Trebižat, *N* = 39	9	16	9	5					7	21	11						2	15	19	3				2	22	11	4				
Lower Matica, *N* = 7		4	1	2						2	3	2						3	3	1					1	3	3				
Total, *N* = 46	**9**	**20**	**10**	**7**					**7**	**23**	**14**	**2**					**2**	**18**	**22**	**4**				**2**	**23**	**14**	**7**				
	**Total vertebrae**				**Abdominal vertebrae**		**Caudal vertebrae**				**Predorsal vertebrae**			**Vertebral formulae**																	
	**41**	**42**	**43**	**44**	**24**	**25**	**17**	**18**	**19**	**20**	**14**	**15**	**16**	**24+17**	**24+18**	**24+19**	**24+20**	**25+17**	**25+18**	**25+19**											
*S. tenellus*, *N* =25		4	18	3	15	10	1	11	11	2		21	4		3	**10**	2	1	**8**	1											
*S. microlepis* phenotype 1, *N* =43	1	24	23	2	29	21	6	34	11			39	11	1	**19**	9		5	**14**	2											
*S. microlepis* phenotype 2, *N* =44		4	25	4	27	10	1	11	24	1	5	31	1		3	**21**	1	1	**8**	3											

**Table 4.** Morphometric data of *Squalius microlepis* phenotypes in two size classes.

	Phenotype 1, *N* =20				Phenotype 1, *N* =27				Phenotype 2, *N* =15				Phenotype 2, *N* =31			
	min	max	m	sd	min	max	m	sd	min	max	m	sd	min	max	m	sd
SL, mm	61.1	121.7	94.3		125.3	223.4	178.4		72.0	128.0	98.5		135.7	255.7	182.3	
Maximum body depth (% SL)	21.1	24.6	22.3	0.8	22.8	26.7	24.2	1.1	21.4	24.7	22.7	1.1	20.9	25.1	23.0	1.1
Depth of caudal peduncle (% SL)	9.1	10.4	9.7	0.4	9.1	10.6	9.9	0.5	9.5	10.8	10.2	0.4	9.5	11.0	10.3	0.4
Depth of caudal peduncle (% length of caudal peduncle)	45.7	56.0	50.9	3.4	46.1	56.6	51.1	3.0	45.9	55.8	51.1	3.3	46.0	59.5	52.6	3.5
Maximum body width (% SL)	11.8	15.3	13.5	0.9	12.4	16.0	13.6	0.9	13.1	16.1	13.9	0.8	14.0	17.7	15.6	0.8
Caudal peduncle width (% SL)	7.6	10.3	8.7	0.7	7.2	9.7	8.2	0.7	7.8	10.2	8.8	0.6	8.6	11.1	9.6	0.5
Predorsal length (% SL)	57.0	59.7	58.5	0.9	56.1	60.5	58.2	1.1	54.3	58.5	56.3	1.3	54.5	58.3	56.4	1.2
Postdorsal length (% SL)	30.4	34.6	32.2	1.3	30.3	35.8	32.5	1.5	32.8	35.4	34.4	0.8	33.2	35.8	34.5	0.7
Prepelvic length (% SL)	52.5	57.9	54.6	1.4	50.9	59.4	55.0	2.5	50.7	54.3	52.0	1.0	49.5	54.2	51.8	1.2
Preanal length (% SL)	69.9	78.7	73.6	2.2	70.4	78.7	73.8	2.1	70.9	73.5	72.3	0.8	70.3	74.4	72.1	1.2
Pectoral – pelvic-fin origin length (% SL)	22.9	27.6	25.0	1.3	24.8	29.3	26.5	1.2	23.8	28.5	25.9	1.3	24.0	29.1	26.4	1.3
Pelvic – anal-fin origin length (% SL)	17.4	20.3	18.8	0.9	18.3	23.6	20.1	1.2	19.2	23.2	20.9	1.2	19.0	22.4	21.1	0.9
Length of caudal peduncle (% SL)	16.9	20.9	19.2	1.3	17.3	21.8	19.4	1.1	19.3	20.9	20.0	0.6	17.1	21.6	19.7	1.1
Dorsal-fin base length (% SL)	9.4	11.5	10.9	0.6	9.7	13.3	11.4	0.8	9.2	12.6	11.0	0.9	9.9	13.6	11.5	0.9
Dorsal fin depth (% SL)	15.4	18.9	17.2	1.0	13.9	17.7	15.6	0.9	14.9	18.9	16.9	1.2	14.3	17.1	15.4	0.7
Anal-fin base length (% SL)	8.8	11.5	10.2	0.8	8.1	12.4	10.1	1.0	9.7	11.4	10.6	0.4	9.6	12.3	10.8	0.6
Anal fin depth (% SL)	12.2	15.8	13.2	1.1	10.2	14.0	12.0	0.6	10.7	14.5	12.5	0.9	10.4	12.7	11.7	0.6
Pectoral fin length (% SL)	16.9	19.1	18.0	0.7	15.4	19.1	17.4	1.0	16.5	20.0	17.7	1.0	14.7	18.7	17.0	0.9
Pelvic fin length (% SL)	13.0	15.9	14.4	0.7	13.3	15.2	14.0	0.6	13.3	15.5	14.4	0.6	12.6	15.2	13.8	0.7
Head length (% SL)	29.7	32.6	31.2	0.9	29.0	33.7	30.9	1.5	25.9	29.6	27.8	1.0	25.4	28.2	27.1	0.8
Head length (% body depth)	132.7	148.3	140.4	5.3	114.0	142.6	126.8	6.6	109.2	138.4	122.9	8.0	108.5	126.1	117.8	5.0
Head depth at nape (% SL)	16.3	19.0	17.7	0.6	16.9	20.3	18.0	1.0	16.2	17.8	16.9	0.4	15.8	18.4	17.0	0.7
Head depth at nape (% HL)	52.4	61.2	56.8	2.3	54.3	60.9	58.6	1.7	58.2	64.9	60.7	2.1	59.4	66.8	63.0	2.1
Maximum head width (% SL)	13.5	15.9	14.6	0.6	13.6	15.4	14.5	0.5	13.2	14.4	13.9	0.4	13.1	15.6	14.3	0.6
Maximum head width (% HL)	43.7	50.4	46.8	1.8	43.1	52.3	47.3	2.4	45.8	54.2	49.9	2.2	48.3	57.9	52.8	2.3
Snout length (% SL)	8.2	9.9	9.1	0.5	8.1	10.6	9.3	0.6	7.7	9.2	8.3	0.4	7.8	9.0	8.4	0.3
Snout length (% HL)	27.2	30.8	29.0	1.4	27.5	32.4	30.3	1.3	27.4	32.6	29.8	1.6	29.2	33.6	31.2	1.2
Eye horizontal diameter (% SL)	6.2	7.9	7.1	0.6	4.6	6.4	5.1	0.5	5.0	6.6	5.8	0.5	4.1	5.4	4.7	0.3
Eye horizontal diameter (% HL)	20.0	25.7	22.8	2.0	14.1	19.5	16.7	1.3	18.0	23.4	20.7	1.6	15.0	20.3	17.5	1.4
Eye horizontal diameter (% interorbital width)	61.8	86.5	75.7	8.1	46.8	72.4	55.2	5.6	56.8	69.5	63.0	4.8	44.0	61.0	51.6	4.9
Postorbital distance (% HL)	50.4	54.2	51.9	1.2	50.0	58.1	55.1	1.7	51.9	57.4	54.0	1.3	51.8	56.9	54.5	1.4
Interorbital width (% SL)	8.8	10.3	9.4	0.4	8.3	10.1	9.3	0.5	8.8	9.6	9.1	0.3	8.5	9.8	9.2	0.3
Interorbital width (% HL)	27.2	32.6	30.2	1.4	26.7	32.9	30.3	1.3	31.1	35.4	32.8	1.4	30.4	36.6	34.1	1.2
Length of upper jaw (% HL)	28.0	32.5	30.1	1.1	27.1	34.1	30.8	1.7	27.2	30.0	29.1	0.8	28.3	33.0	29.6	1.0
Length of upper jaw (% SL)	8.6	10.1	9.4	0.4	8.4	11.4	9.4	0.7	7.7	8.5	8.1	0.3	7.4	8.9	8.0	0.3
Length of lower jaw (% SL)	11.4	13.6	12.6	0.5	11.3	13.5	12.4	0.6	10.0	11.1	10.4	0.4	9.3	10.7	10.1	0.3
Length of lower jaw (% HL)	35.9	43.9	40.5	1.7	37.9	43.7	40.3	1.5	35.9	39.7	37.5	1.2	34.8	40.5	37.3	1.0
Length of lower jaw (% interorbital width)	122.3	146.7	134.0	5.3	121.4	142.7	133.5	5.3	109.1	120.9	114.5	3.7	99.7	119.7	109.4	3.4
Length of lower jaw (% depth of operculum)	111.7	128.6	118.7	4.9	107.3	125.9	117.4	4.7	98.9	111.4	103.9	3.8	89.1	114.1	101.9	5.3
Maximum cranial width (% cranium roof length)	63.9	76.8	69.4	4.4	64.0	73.8	68.0	2.6	67.7	77.2	71.6	2.9	69.0	81.6	73.9	4.9
Supraethmoid width (% cranium roof length)	20.0	25.7	23.0	1.8	21.1	26.5	24.1	1.7	20.0	26.1	24.4	1.6	20.5	26.5	23.4	1.4
Length of lower jaw (% maximum cranial width)	95.2	110.9	102.7	5.6	97.9	115.4	106.6	4.9	81.7	94.2	89.5	3.7	81.3	100.9	89.7	4.1
Depth of operculum (% HL)	32.1	36.4	34.1	1.3	32.0	38.7	34.5	1.7	34.0	38.7	36.1	1.7	32.1	41.5	36.6	2.1
RATIOS:																
Interorbital width/eye horizontal diameter	1.2	1.6	1.3	0.2	1.4	2.1	1.8	0.2	1.4	1.8	1.6	0.1	1.6	2.3	2.0	0.2
Snout length/eye horizontal diameter	1.1	1.5	1.3	0.2	1.4	2.2	1.8	0.2	1.2	1.6	1.4	0.1	1.5	2.1	1.8	0.2
Head depth at nape/eye horizontal diameter	2.2	2.9	2.5	0.2	2.8	4.3	3.5	0.3	2.6	3.4	3.0	0.3	3.1	4.2	3.6	0.3
Head length/caudal peduncle depth	2.9	3.5	3.2	0.2	2.8	3.5	3.1	0.2	2.4	3.0	2.7	0.2	2.3	2.8	2.6	0.1
Length of caudal peduncle/caudal peduncle depth	1.8	2.2	2.0	0.1	1.8	2.2	2.0	0.1	1.8	2.2	2.0	0.1	1.7	2.2	1.9	0.1
Length of lower jaw/caudal peduncle depth	1.1	1.4	1.3	0.1	1.1	1.4	1.3	0.1	1.0	1.1	1.0	0.1	0.9	1.1	1.0	0.0
Pectoral fin length/pectoral – pelvic-fin origin distance	0.6	0.8	0.7	0.1	0.6	0.8	0.7	0.0	0.6	0.8	0.7	0.1	0.6	0.7	0.6	0.0
Predorsal length/head length	1.8	1.9	1.9	0.0	1.7	2.0	1.9	0.1	1.9	2.1	2.0	0.1	2.0	2.2	2.1	0.1

**Table 5.** Morphometric and meristic data of *Squalius microlepis* specimens classified in separate set of analyses (see text for explanations).

Collection	NMW 49428	NMW 49427	NMW 49423:1	NMW 49423:2	NMW 49424	NMW 49425	NMW 49426	NMW 49228:1	NMW 49228:2	MNCN_ICTIO 292.129–292.136	MNCN_ICTIO 292.129-292.136
Stated locality	Lago di Dusino presso Imotsky	Narenta	Vrgoraz [see Jessero]	Vrgoraz [see Jessero]	Vrgoraz [see Jessero]	Vrgoraz	Vrgoraz [see Jessero]	Zara	Zara	Tihaljina	Tihaljina
Specimen	A	B	C	D	E	F	G	H	I	J	K
SL, mm	168.07	140.06	122.52	276.08	269.1	177.98	193.81	165.84	205.13	127.05	149.75
Maximum body depth (% SL)	26.26	21.58	23.20	23.70	22.84	24.74	21.21	24.57	27.75	23.74	23.88
Depth of caudal peduncle (% SL)	11.42	10.35	11.40	10.53	10.62	10.92	9.78	9.86	10.95	9.82	10.09
Depth of caudal peduncle (% length of caudal peduncle)	56.06	51.53	60.40	58.81	55.68	54.27	50.71	50.25	56.83	49.04	47.85
Body width at dorsal-fin origin (% SL)	13.73	11.82	13.64	12.26	11.91	13.02	13.84	12.75	15.37	13.59	16.05
Caudal peduncle width (% SL)	9.64	7.65	9.72	7.38	7.08	8.39	9.40	8.47	8.98	8.26	8.97
Predorsal length (% SL)	58.22	56.55	58.52	57.46	56.85	57.03	58.17	59.35	59.16	55.58	55.26
Postdorsal length (% SL)	33.30	36.58	32.59	33.75	34.40	35.35	34.34	31.08	32.57	34.07	33.92
Prepelvic length (% SL)	52.46	53.59	52.08	55.14	54.83	52.25	53.00	56.22	54.03	54.55	52.73
Preanal length (% SL)	74.87	72.84	73.87	75.86	72.83	74.96	74.22	75.61	73.37	72.61	72.33
Pectoral – pelvic-fin origin length (% SL)	25.97	24.90	24.38	26.80	26.93	27.69	27.77	26.47	24.59	27.63	24.89
Pelvic – anal-fin origin length (% SL)	22.37	18.76	20.66	22.07	20.60	23.49	22.11	20.15	21.19	19.87	19.97
Length of caudal peduncle (% SL)	20.38	20.08	18.88	17.91	19.08	20.11	19.29	19.63	19.28	20.03	21.09
Dorsal-fin base length (% SL)	10.22	10.55	10.10	10.86	11.56	10.41	10.12	11.29	12.12	11.69	11.71
Dorsal fin depth (% SL)	17.56	16.12	16.50	15.81	15.22	15.06	15.53	14.96	14.20	15.93	16.39
Anal-fin base length (% SL)	8.91	10.37	10.55	9.34	10.24	10.39	9.20	10.34	10.95	10.38	11.52
Anal fin depth (% SL)	11.23	12.78	11.57	10.61	12.15	10.60	11.80	11.82	12.61	10.83	11.81
Pectoral fin length (% SL)	16.01	18.14	17.38	17.25	18.12	14.25	14.64	18.31	18.19	16.98	18.01
Pelvic fin length (% SL)	13.74	13.65	13.86	13.35	13.71	12.20	12.49	15.24	15.43	12.96	14.03
Head length (% SL)	27.89	28.67	29.61	29.34	29.49	27.22	27.01	30.37	30.53	28.96	29.47
Head length (% body depth)	106.18	132.82	127.66	123.80	129.12	110.04	127.32	123.61	110.01	122.02	123.41
Head depth at nape (% SL)	17.86	16.92	17.52	18.90	18.31	16.91	18.50	19.09	19.24	16.68	17.32
Head depth at nape (% HL)	64.05	59.03	59.15	64.42	62.06	62.11	68.49	62.85	63.03	57.58	58.78
Head depth through eye (% HL)	43.82	39.78	40.96	47.36	43.79	40.25	47.00	39.25	42.33	39.76	41.58
Maximum head width (% SL)	13.71	12.82	12.59	14.18	13.42	13.41	14.32	13.53	14.17	13.40	14.31
Maximum head width (% HL)	49.18	44.71	42.53	48.35	45.50	49.25	53.04	44.55	46.42	46.25	48.56
Snout length (% SL)	8.67	8.00	8.43	9.18	8.20	7.92	8.19	8.80	8.82	8.32	8.83
Snout length (% HL)	31.11	27.90	28.47	31.30	27.81	29.10	30.32	28.97	28.90	28.72	29.96
Eye horizontal diameter (% SL)	4.19	6.31	6.18	4.37	4.86	4.53	4.27	5.69	5.57	5.31	4.77
Eye horizontal diameter (% HL)	15.02	22.02	20.87	14.90	16.47	16.64	15.80	18.74	18.25	18.34	16.18
Eye horizontal diameter (% interorbital width)	46.25	77.89	69.96	45.91	55.36	46.86	45.62	63.53	60.99	60.70	52.31
Postorbital distance (% HL)	57.33	55.29	52.59	59.59	56.90	56.97	58.54	55.17	54.46	54.18	54.82
Interorbital width (% SL)	9.06	8.10	8.83	9.52	8.77	9.66	9.35	8.96	9.14	8.75	9.12
Interorbital width (% HL)	32.47	28.27	29.82	32.46	29.75	35.50	34.64	29.50	29.93	30.22	30.93
Length of upper jaw (% HL)	30.51	24.66	27.73	32.51	32.49	29.37	29.27	30.91	32.35	29.35	29.05
Length of upper jaw (% SL)	8.51	7.07	8.21	9.54	9.58	8.00	7.90	9.39	9.88	8.50	8.56
Length of lower jaw (% SL)	10.37	10.42	10.74	12.50	12.26	10.20	10.33	12.69	12.28	11.14	11.39
Length of lower jaw (% HL)	37.19	36.34	36.27	42.59	41.58	37.46	38.25	41.79	40.21	38.45	38.64
Length of lower jaw (% interorbital width)	114.52	128.55	121.63	131.23	139.77	105.52	110.42	141.66	134.36	127.25	124.91
Length of lower jaw (% depth of operculum)	104.31	96.24	112.48	107.78	111.49	100.33	96.95	113.11	100.12	107.36	101.49
Cranium width between margins of pterotics (% L cranium roof length)	64.68	70.41	68.90	72.54	72.41	78.53	78.75	75.04	73.82	75.76	70.96
Cranium width between margins of sphenotics (% cranium roof length)	59.57	57.79	61.02	61.22	60.14	64.77	69.80	62.63	59.88	62.32	57.52
Cranium width between margins of supraethmoid (% cranium roof length)	28.40	22.60	25.23	22.42	27.38	23.54	27.87	24.64	25.54	26.77	23.51
Cranium width between margins of supraethmoid (% cranium width between margins of pterotics)	43.91	32.10	36.62	30.91	37.81	29.97	35.39	32.83	34.60	35.34	33.13
Length of lower jaw (% cranium width between margins of pterotics)	95.66	89.90	89.58	98.21	105.13	85.53	85.89	102.38	99.68	94.71	94.15
Depth of operculum (% HL)	35.65	37.76	32.25	39.52	37.29	37.34	39.45	36.95	40.16	35.82	38.07
RATIOS:											
Interorbital width/eye horizontal diameter	2.16	1.28	1.43	2.18	1.81	2.13	2.19	1.57	1.64	1.65	1.91
Snout length/eye horizontal diameter	2.07	1.27	1.36	2.10	1.69	1.75	1.92	1.55	1.58	1.57	1.85
Head depth at nape/eye horizontal diameter	4.26	2.68	2.83	4.32	3.77	3.73	4.33	3.35	3.45	3.14	3.63
Head length/caudal peduncle depth	2.44	2.77	2.60	2.79	2.78	2.49	2.76	3.08	2.79	2.95	2.92
Length of caudal peduncle/caudal peduncle depth	1.78	1.94	1.66	1.70	1.80	1.84	1.97	1.99	1.76	2.04	2.09
Length of lower jaw/caudal peduncle depth	0.91	1.01	0.94	1.19	1.15	0.93	1.06	1.29	1.12	1.13	1.13
Pectoral fin length/pectoral – pelvic-fin origin distance	0.62	0.73	0.71	0.64	0.67	0.51	0.53	0.69	0.74	0.61	0.72
Predorsal length/head length	2.09	1.97	1.98	1.96	1.93	2.10	2.15	1.95	1.94	1.92	1.88
COUNTS: Scales in lateral series	71	76	68	74	67	66	74	72	71	72	70
Total lateral-line scales	68	74	67	73	66	64	72	70	69	71	70
Scales above lateral line	14	15	14	14	14	13	16	15	14	15	14
Scales below lateral line	7	6	5	6	6	6	7	6	6	6	6
Gill rakers	15	14	15	15	14	12	13	15	15	16	15
Number of predorsal vertebrae	15	15	15	15	15	15	15	15	15	15	15
Number of abdominal vertebrae	24	24	25	24	25	25	25	25	25	25	24
Number of caudal vertebrae	19	19	17	18	17	18	18	17	17	18	19
Total vertebrae	43	43	42	42	42	43	43	42	42	43	43
Difference between abdominal and caudal counts	5	5	8	6	8	7	7	8	8	7	5

For statistical processing of data, to partly remove the size component from the shape measures, we used: 1) all individual morphometric character measurements standardised following Elliott et al. (1995) and 2) as proportional measurements (as in Tables 2, 3). Taking into account the relatively small sample sizes and the lack of information about the distribution of variables, nonparametric statistic tests (Mann-Whitney and Kruskal-Wallis) were used. Multivariate data analyses included forward stepwise discriminant function analysis (DFA) and cluster analysis (CA; using the unweighted pair-group average method with Euclidean distance). When analysing variables measured at different scales, z-transformation was used. The statistical analyses were performed using Microsoft Excel, Statistica 6.0 (Statistic for Windows. StatSoft) and SPSS Statistics V23.0 (IBM SPSS).

Abbreviations used:

**MNCN_ICTIO** Ichthyology Collection, Museo Nacional de Ciencias Naturales, Madrid, Spain;

**MZUF** Universita di Firenze, Museo Zoologico e Historia Naturale de la Specola, Firenze, Italy;

**NMW** Naturhistorisches Museum, Wien, Austria;

**PZC** Collection of P. Zupančič, Dolsko, Slovenia;

**ZISP** Zoological Institute, Russian Academy of Sciences, St. Petersburg, Russia; HL, head length;

**SL** standard length;

**s. l.** sensu lato;

**s. str.** sensu stricto.

## Results

The data presented in Tables 2, 3 and osteological and sensory canal examinations confirmed a traditional concept of *S. microlepis* and *S. tenellus* (Bănărescu and Herzig-Straschil 1998; Kottelat and Freyhof 2007) as a morphologically distinct group different from other *Squalius* species in Europe. This group can be clearly distinguished by having small scales (64–95 in total lateral series and 58–83 in total lateral line) and a reduced or lacking 5^th^ infraorbital.

**Figure 2.**
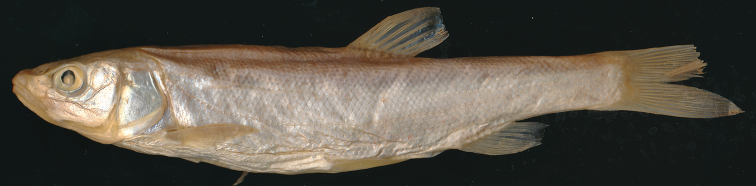
*Squalius tenellus*, NMW 16001, lectotype, 122.1 mm SL, ‘Livno’.

*Squalius tenellus* (Fig. 2) has markedly smaller scales than *S. microlepis* s. l. and, respectively, higher numbers of total lateral-line scales, total scales in lateral series and scales above (to the dorsal-fin origin) and below (to the pelvic-fin origin) the lateral line. For *S. tenellus*, ranges of these character states are as follows: 76–95 (80 in lectotype) scales in lateral series, 68–83 (78 in lectotype) in lateral line, 17–20 (19 in lectotype) above lateral line, and 7–10 (9 in lectotype) above lateral line. These numbers are different from those commonly published based on data of Bănărescu and Herzig-Straschil (1998: 420); this may be due to a different method of counting. *Squalius tenellus* can be further distinguished by an often slightly incomplete, interrupted or deformed lateral line and scales somewhat irregularly placed on the back and flanks; these traits have not been found in *S. microlepis*.

**Figure 3.**
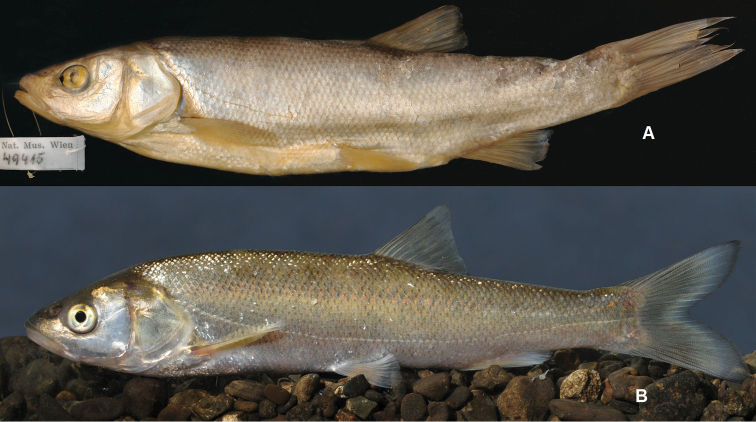
*Squalius microlepis***A** NMW 49415, lectotype, 150.8 mm SL, “Imosky” **B** phenotype 1: alive specimen, MNCN_ICTIO 296.096-296.097, 147.6 mm SL, Bosnia and Herzegovina: Krenica Lake at Drinovci.

**Figure 4.**
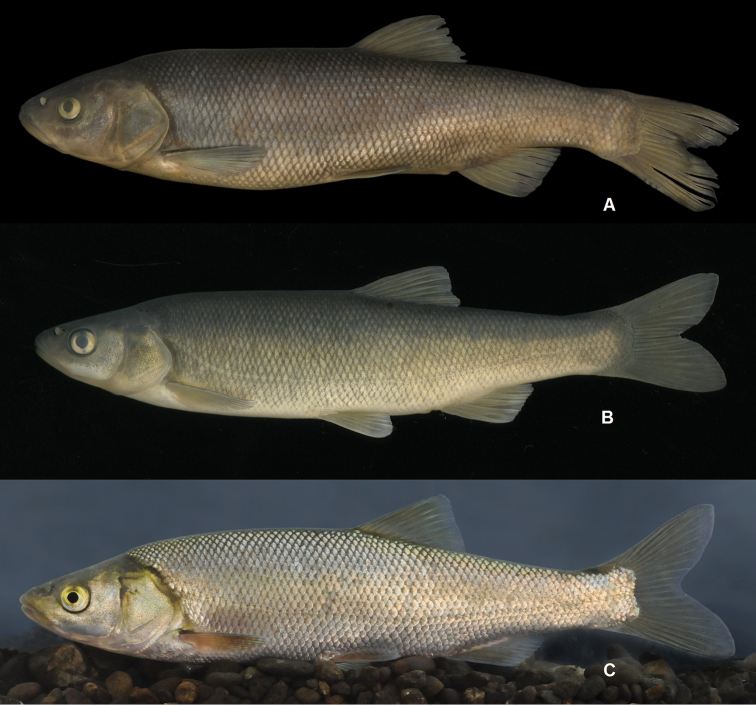
*Squalius microlepis* phenotype 2 **A** SMNH 443, 255.7 mm SL, Bosnia and Herzegovina: Tihaljina River at Tihaljina **B** MNCN_ICTIO 294.472-294.473, 140.1 mm SL, Bosnia and Herzegovina: Trebižat River at Grabovnik-Vašarovići **C** alive specimen, ZISP 54994, 147.2 mm SL, Bosnia and Herzegovina: Matica (Vrljika) River at Drinovci.

**Figure 5.**
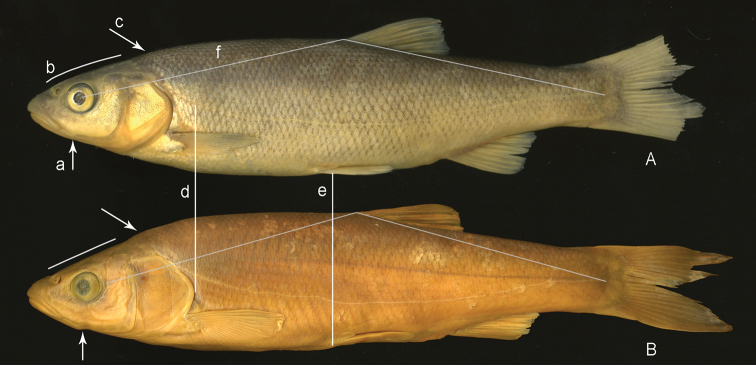
Lateral view to show characters superficially distinguishing phenotypes of *Squalius microlepis* s. l. **A***Squalius microlepis* phenotype 2: MNCN_ICTIO 294.784-294.800, 128.0 mm SL (Tihaljina) **B***Squalius microlepis* phenotype 1: NMW 12729-32, 119.5 mm SL (‘Imotski’). Key: Arrow a – posterior end of lower jaw, line b – upper head profile, arrow c – body profile just behind head; vertical d – shorter head in *S. microlepis* phenotype 2, vertical e – shorter prepelvic distance in *S microlepis* phenotype 2; line f – dorso-hypural distance if reported forward.

**Figure 6.**
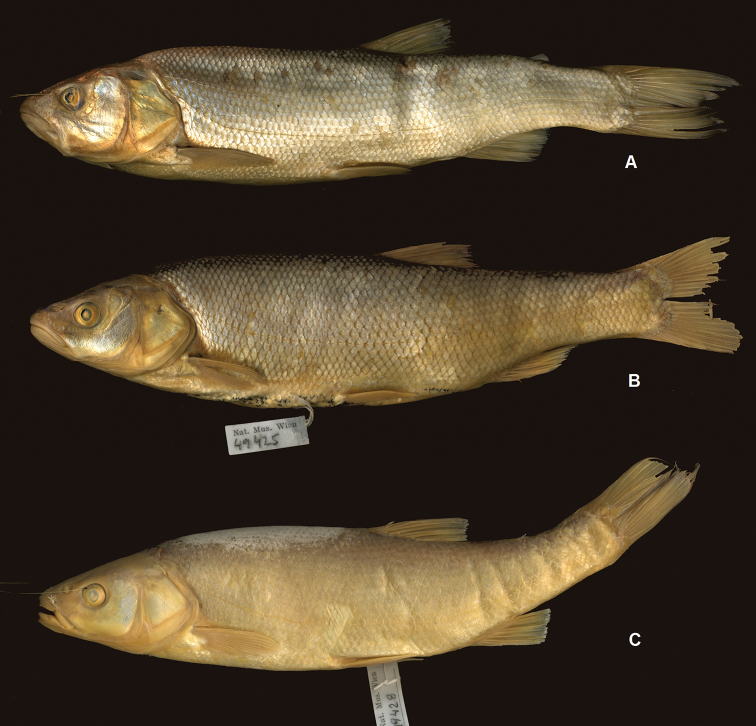
*Squalius microlepis* phenotype 1, karst systems near Vrgorac **A** phenotype 1, NMW 49423, 276.1 mm SL, ‘Vergoraz [See Jessero]’ (specimen E) **B** phenotype 2, NMW 49425, 178 mm SL, ‘See bei Gradač & Vrgorač’ (specimen C); **C**, NMW 49428, 165.8 mm SL, ‘Lago di Dusino’, intermediate between phenotypes 1 and 2 (specimen A: external appearance as in phenotype 2 but 15 gill rakers as in phenotype 1).

An examination of the entire set of *Squalius microlepis* examined specimens (Tables 2–5, Figs 3–6) revealed a number of character states that allow to distinguish two phenotypes: phenotype 1 representing *S. microlepis* s. str. as defined by its lectotype (Fig. 3) and phenotype 2 as represented in Fig. 4.

The first step morphological analyses and comparisons excluded specimens A to K as specified in Table 1 and the primary data presented in Table 4. The reasons were as follows:

1 uncertainty of the localities

– “Lago di Dusino” (specimen A); we suppose the locality is not ‘near Imotski’ but the Dusina Polje (Lake of Dusina) formed by some large karstic springs at the village Dusina south of Vrgorac and immediately adjacent to Polje Jezero;

– “Narenta” (B); *S. microlepis* is not reliably known from the main stream of the Neretva as discussed below;

– “Zara” (H, I), an NMW historic sample, labelled as ‘Zara’ (Italian name for Zadar), a locality not known for *S. microlepis*-like species and 200 km outside the known range of *S. microlepis* s. str. (probable mislabelling of the sample is discussed below);

2 a high morphological heterogeneity of the sample from karst systems at Vrgorac (C to G); karstic poljes near Vrgorac are geographically distant from the Imotski system though connected to the Tihaljina-Trebižat system; this area is of a special historical importance because no other specimens are extant in collections to our knowledge neither we were able to collect this fish in karts systems near Vrgorac;

3 specimens J and K are the only ones similar to phenotype 1 among the numerous samples of the Tihaljina-Trebižat phenotype 2.

The second step was to run separate statistical analyses for identification of these specimens.

### Size-related variability in two phenotypes of *S. microlepis*

Table 4 contains data on a comparison of smaller-sized (SL < 130 mm) and larger-sized (SL > 130 mm) specimens per phenotype. Significantly size-related (p < 0.01000) are 18 characters in *S. microlepis* phenotype 1 and 22 characters in *S. microlepis* phenotype 2. Shared size-related characters are as follows: dorsal fin depth (% SL), anal fin depth (% SL), head length (% SL), head length (% body depth), head depth at nape (% HL), snout length (% HL), eye horizontal diameter (% SL), eye horizontal diameter (% HL), eye horizontal diameter (% interorbital width), interorbital width/eye horizontal diameter, snout length/eye horizontal diameter, head depth at nape/eye horizontal diameter, pectoral fin length/pectoral – pelvic-fin origin distance, predorsal length/head length. Head depth at nape and snout length increase with size while anal- and dorsal-fin depth, head length, and eye diameter decrease.

### Difference between two phenotypes of *S. microlepis*

The two phenotypes are readily distinguished (phenotype 1 vs. phenotype 2; external characters on an example of middle-sized specimens see Fig. 5; Tables 2–4) by the following combinations of character states:

1 number of gill rakers: (13)14–16 (15 in lectotype of *S. microlepis*), mean 15.0 vs. 11–14, mean 12.6;

2 total vertebrae: commonly 42 (24+18 or 25+17) and 43 (25+18) vs. commonly 43 (24+19);

3 dorso-hypural distance: commonly falling behind the posterior eye margin (at a considerable distance from the eye margin in large-sized specimens as can be also seen in Kottelat and Freyhof (2007: figure on page 269) vs. commonly falling into the middle or posterior half of the eye when reported forward;

4 the back: usually a well pronounced discontinuity behind the head (even in small-sized individuals), a straightened back profile and the maximum body depth located just behind the head vs. smoothly convex lacking a prominent hump behind the head and the maximum body depth located at or slightly in front of the dorsal-fin origin;

5 maximum body depth: the body deepest at a vertical closer to the head than to the dorsal-fin origin and, respectively, maximum body depth exceeds 1.05–1.20 times body depth at the dorsal-fin origin vs. about equal to body depth at the dorsal-fin origin;

6 length of lower jaw (% interorbital width): 121–155% (mean 134.5%) vs. 100–121% (mean 111%); length of lower jaw (% cranium width) 95–118% (mean 105% vs. 81–101% (mean 90%);

7 head length (% SL): 29–34% (mean 31%) vs. 25–30% (mean 27%); the ranges do not overlap in larger-sized specimens (SL > 130 mm; Table 3);

8 head depth at nape (% HL) in larger-sized specimens (SL > 130 mm; Table 3): 54–61% (mean 59%) vs. 59–69% (mean 63%);

9 the upper head profile: straight vs. commonly slightly convex behind the eyes.

Besides these characters, Fig. 5B illustrates a specimen of *S. microlepis* phenotype 1 having the upper jaw not projecting beyond the lower jaw, the lower jaw-quadrate junction lying on the vertical through the middle of the eye, and a prominent ‘angle’ formed by the posterior end of the lower jaw; and the mouth cleft is long, straight and oblique. Phenotype 2 (Fig. 5A) is commonly characterised by the upper jaw clearly projecting beyond the lower jaw and including the tip of the lower jaw; the lower jaw-quadrate junction located about at a vertical through or slightly in front of the anterior margin of the pupil; the lower jaw posterior end not forming a prominent angle; and the mouth cleft slightly curved and more horizontal.

### Statistical analyses

Comparison of the two phenotypes of *S. microlepis*

**Figure 7.**
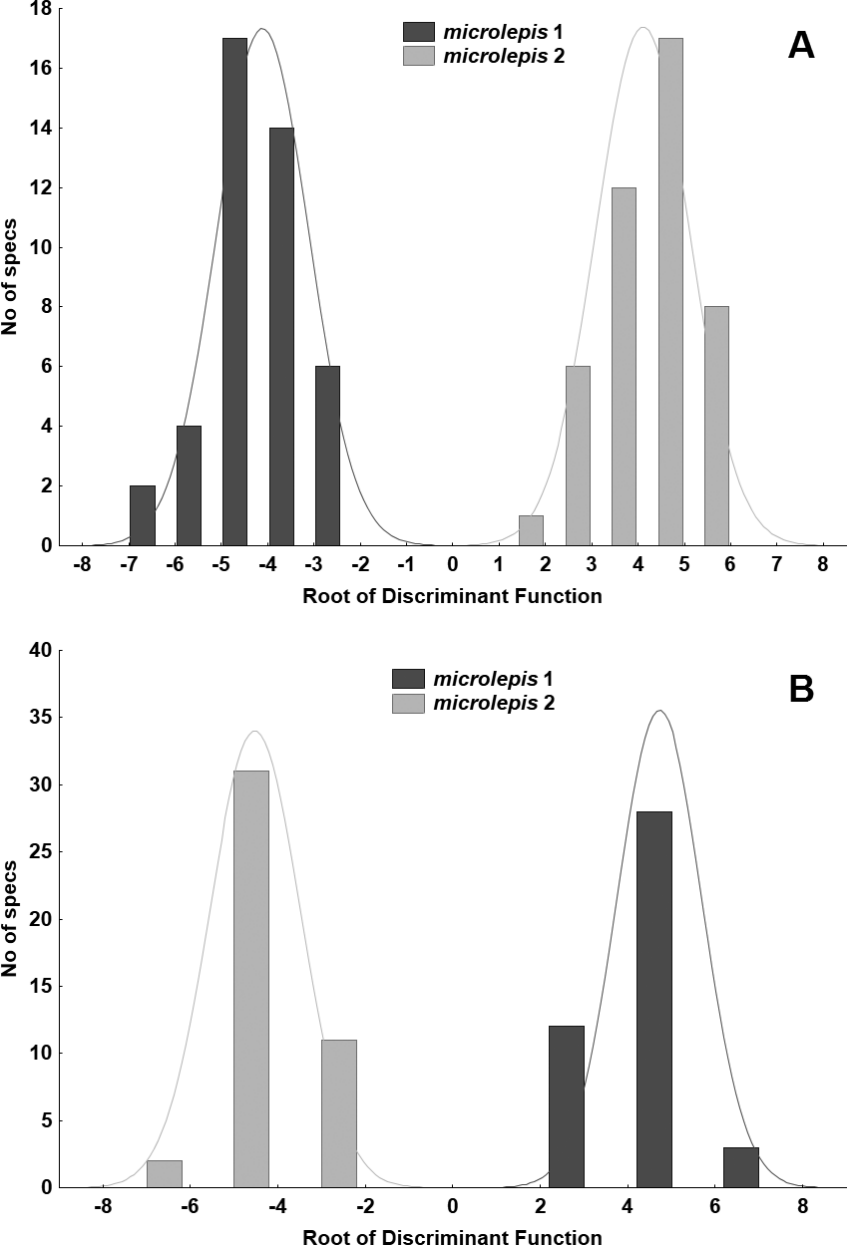
DFA performed for two combined samples of *Squalius microlepis* phenotype 1 and phenotype 2 **A** based on 32 standardised direct measurements and 12 counts **B** based on 52 proportional measurements (as in Table 2) and counts. Specimens A–K in Table 5 not included.

**I** A DFA based on counts and standardised direct measurements (Fig. 7A) support 100% discrimination for both groups. DFA statistics values are as follows: Wilks’ Lambda 0.5525, *appr. F* (19.64) = 60.297, *P* < 0.0000. In this analysis, the lower jaw length, number of gill rakers, head length, upper caudal-fin lobe and maximum head width contribute most to the discrimination between the phenotypes.

**Figure 8.**
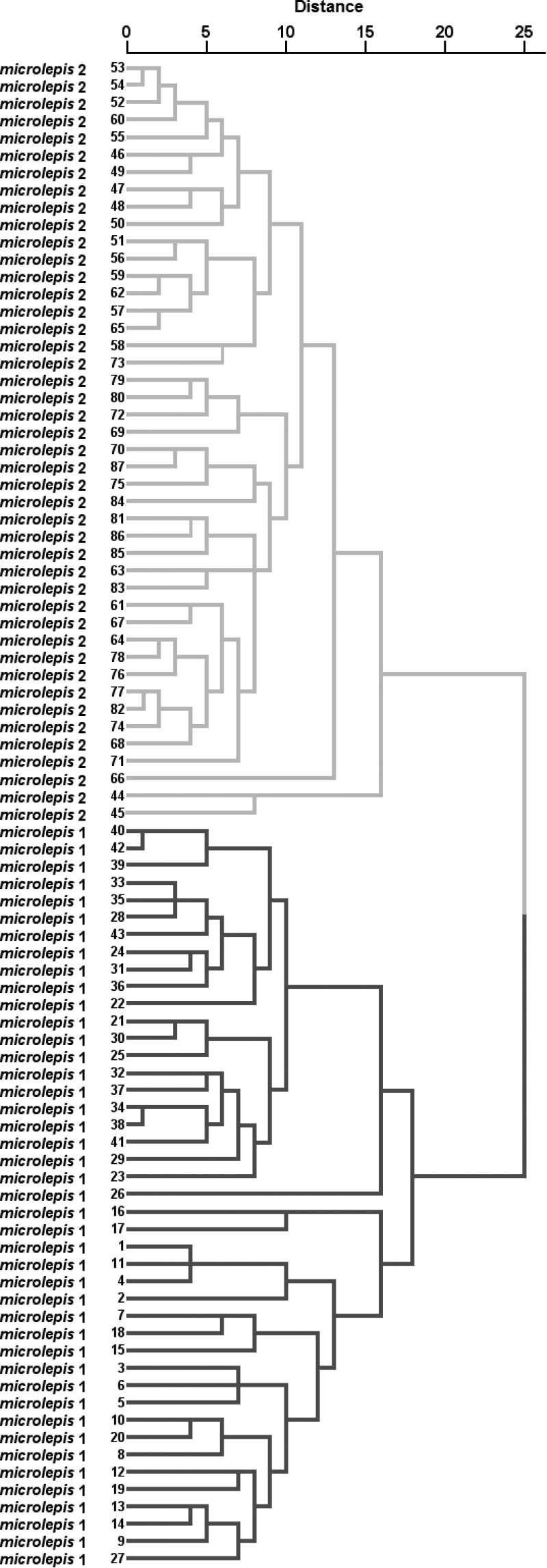
A CA performed for two combined samples of *Squalius microlepis* phenotype 1 and phenotype 2, based on 52 proportional measurements (as in Table 2) and counts. Specimens A–K in Table 5 not included.

**II** A DFA based on counts and relative measurements (as in Table 2) (Fig. 7B) also support 100% discrimination for both groups (Wilks’ Lambda 0.04411, *appr. F* (23.63) = 59.369, *P* < 0.0000), and the most contributing characters are the number of gill rakers, interorbital width (% HL), ethmoid width (% pterotic cranial width), prepelvic length (% SL), and head length (% SL). A CA run for the same set of characters support perfect clusters into two groups (Fig. 8).

Taken together, these two analyses based on differently approached characters, clearly support the primary observations on most influential characters for distinguishing the two phenotypes (1, 6, 7 above): gill rakers count, head length, and length of lower jaw.

Classification of selected specimens A–K between the two phenotypes of *S. microlepis*

Character data for specimens A to K are presented in Table 5.

**I** A DFA classification (posterior probabilities and classification functions) based on counts and direct standardised measurements classify these specimens as follows: specimens B, F, and G are identified as phenotype 2 while others as phenotype 1 (Table 6).

**Table 6.** DFA classifications of specimens of *S. microlepis* not identified a priori to phenotype.

	**Based on counts and direct standardised measurements**						**Based on counts and proportional measurements**					
	**Posterior probabilities**			**Classification functions**			**Posterior probabilities**			**Classification functions**		
**Specimen**	***S. tenellus***	***S. microlepis* phenotype 1**	***S. microlepis* phenotype 2**	***S. tenellus***	***S. microlepis* phenotype 1**	***S. microlepis* phenotype 2**	***S. tenellus***	***S. microlepis* phenotype 1**	***S. microlepis* phenotype 2**	***S. tenellus***	***S. microlepis* phenotype 1**	***S. microlepis* phenotype 2**
**A**	0.000312	0.978473	0.021215	6015	6023	6020	0.000000	0.009082	0.990918	151557	151588	151582
**B**	0.000000	0.000000	1.000000	6045	6071	6087	0.000000	0.001598	0.998402	150795	150825	150824
**C**	0.000000	0.999781	0.000219	5865	5900	5891	0.000000	0.999977	0.000023	153984	154039	154019
**D**	0.000000	0.999995	0.000005	6105	6129	6116	0.000000	0.741566	0.258434	152039	152070	152062
**E**	0.000000	1.000000	0.000000	5726	5786	5766	0.000000	1.000000	0.000000	152076	152127	152105
**F**	0.000000	0.000000	1.000000	5771	5799	5833	0.000000	0.000000	1.000000	150471	150500	150535
**G**	0.000000	0.000000	1.000000	5844	5841	5869	0.000000	0.000000	1.000000	149753	149755	149800
**H**	0.000000	1.000000	0.000000	5755	5813	5797	0.000000	1.000000	0.000000	152084	152144	152114
**I**	0.000000	1.000000	0.000000	5839	5900	5883	0.000000	1.000000	0.000000	152894	152945	152909
**J**	0.000000	0.999998	0.000002	5962	5985	5972	0.000000	1.000000	0.000000	151948	151984	151961
**K**	0.000000	0.999294	0.000706	5979	6010	6003	0.000000	0.999997	0.000003	151776	151818	151798

**Figure 9.**
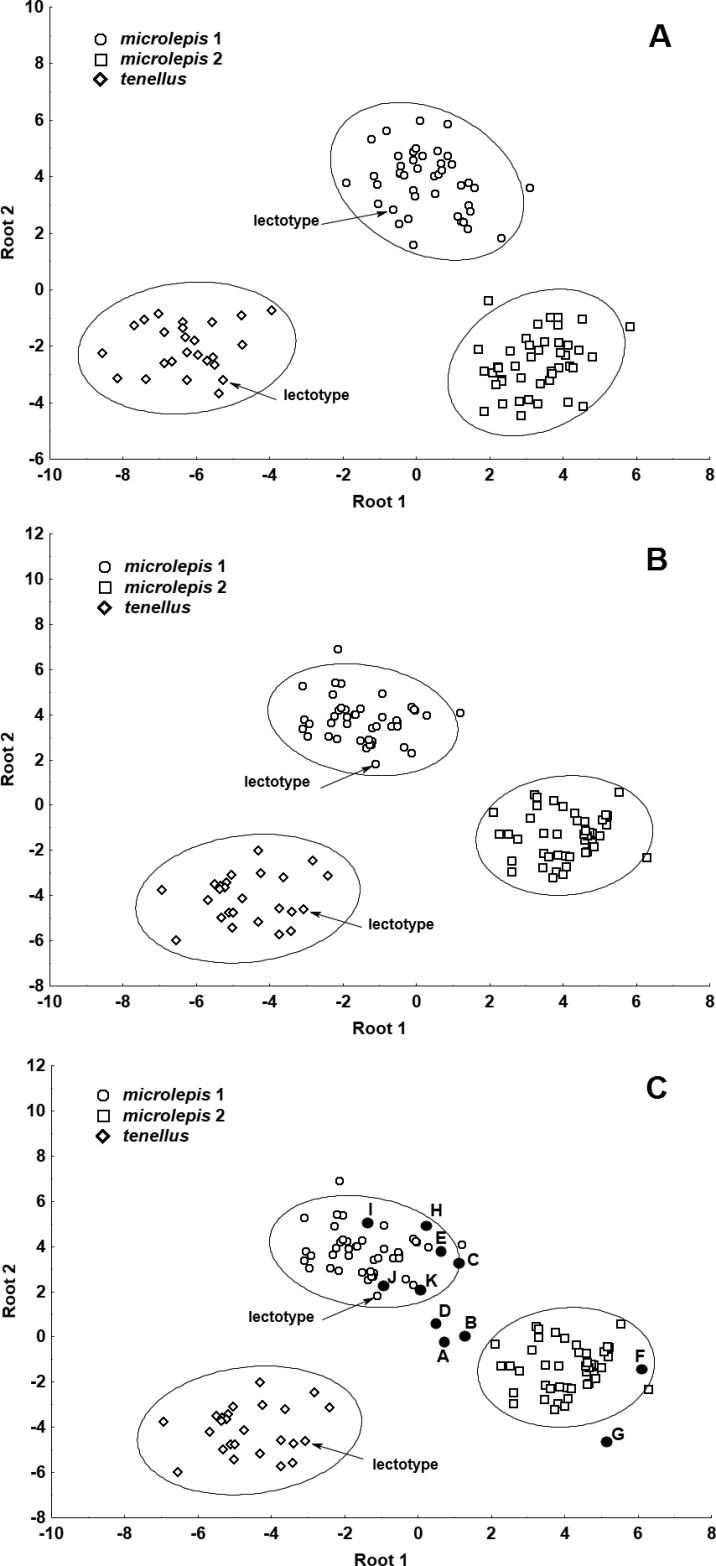
DFA performed for three combined samples, *Squalius tenellus*, *S. microlepis* phenotype 1 and phenotype 2 **A** based on 32 standardised direct measurements and 12 counts (specimens A–K excluded) **B** based on 52 proportional measurements (as in Table 2) and counts (specimens A–K excluded) **C** same analysis as (**B**) but specimens A–K in Table 5 included.

**II** A DFA analyses based on counts and proportional measurements (as in Table 2) (posterior probabilities and classification functions) unambiguously classified specimens C, E, F (Fig. 6A), G–K as phenotype 2. Classification of specimens A and B is variable and classification of specimen D (Fig. 6B) as phenotype 1 is lower than for other specimens. In a DFA scatter plot (Fig. 9C) they are located between phenotypes 1 and 2.

So, the historical NMW sample from poljes at Vrgorac includes both phenotypes of *S. microlepis*. In the Tihaljina-Trebižat kartic system, most specimens were phenotype 2 while two specimens were clearly classified as phenotype 1 (Fig. 9C).

Discrimination of *S. tenellus* and two phenotypes of *S. microlepis*

**I** A DFA performed for three groups of samples (*S. tenellus*, *S. microlepis* phenotype 1 and *S. microlepis* phenotype 2 based on standardised measurements and counts; Fig. 9A) showed a perfect (100%) classification of all three groups (DFA statistics values: Wilks’ Lambda 0.00660, *approx*. *F* (48.172) = 40.519, *P* < 0.0000). The lower jaw length, the number of gill rakers, and the number of scales above the lateral line contribute most to the discrimination between the phenotypes. The closest are two phenotypes of *S. microlepis* and the most distant are *S. tenellus* and *S. microlepis* phenotype 2 (squared Mahalanobis distance equals 52.23712 and 92.95126, respectively).

**II** A DFA performed for the same set of samples but based on the proportional measurements and counts (Fig. 9B) also showed a perfect (100%) classification (DFA statistics values: Wilks’ Lambda 0.00668, *approx*. *F* (44.176) = 44.941, *P*<0.0000). Number of gill rakers, number of scales above the lateral line, number of latera-line scale, maximum head width and maximum cranium width contribute most to the discrimination between the three groups. The closest are two phenotypes of *S. microlepis* and the most distant are *S. tenellus* and *S. microlepis* phenotype 2 (squared Mahalanobis distance equals 57.98632 and 84.69049, respectively). When specimens A to K are included into a DFA analysis, specimens A, B, and D are closely located to each other in the morphological space and intermediate between the two phenotypes (Fig. 9C). Specimens F and G lie within the phenotype 2 while specimens C, E, and H–K lie within the phenotype 1.

## Discussion

### Distribution of *S. microlepis* phenotypes

Ričina-Prološko Blato-Vrljika karst system

The detailed map of this area at the border between Croatia and Bosnia and Herzegovina, its hydrographic networks, position of main discharge gauging stations and supposed groundwater flow directions are presented by Bonacci and Roje-Bonacci (2008: fig. 1) and Bonacci et al. (2013a: fig. 1). We only found individuals of the phenotype 1 in this karst drainage.

All examined specimens from the Ričina-Prološko Blato-Vrljika karst system belong to *S. microlepis* phenotype 1. The NMW labels and acquisition information for the syntypes (lectotype and paralectotypes by Bănărescu and Herzig-Straschil (1998: 417)) say only ‘Imosky, Kroatien (Dalmatien), Heckel Reise 1840’ (as well as some other NMW sample, Table 1) (“Gewässer von Imosky” in the original description (Heckel 1843: 52(1042)). We suppose that the syntypes came, most probably, from Prološko Blato, which is a large swampy region in the north-western part of Imotsko Polje in modern Croatia, named after the town of Imotski (also called Imotski field, or valley, or Imotsko-Bekijsko Polje because the Herzegovinian part of the valley is called Bekija). In 19^th^ century, Prološko Blato was part of the year under water, and just one small part was flooded during the whole year (Proložac, or Prološko Lake). The species also occured in three other lakes close to Prološko Blato: Galipovac, Lokvičić and Knezovića lakes (A. Mikulić, pers. comm. 7 May 2008). For the first time *S. microlepis* was reported in the Vrljika by Katurić (1883) but it is not known how far downstream the Vrljika–Matica River this species was distributed in the past. The Vrljika originates by a spring (izvor) east of Prološko Lake and is at present connected to this lake via canal Sija. The historical NMW sample (1901) from the Vrljika is numerous and contains individuals up to 217 mm SL. Recent samples of *S. microlepis* collected by PZ and DJ in Imotsko Polje are only from Prološko Lake itself, at the inflow of the canal that connects it to the Vrljika. Information from local fishermen (more than ten years ago) indicates that ‘masnica’ had been rarely found in streams of Imotsko Polje but was very abundant in the lake. Further upstream, northwards from Imotsko Polje, *S. microlepis* occured in Ričice Reservoir, a transboundary accumulation lake constructed in the valley of the Ričina River at its confluence with the Vrbica River. It was also found by DJ and PZ in the Ričina River around Posušje (at the village Vir) and in Tribistovo Reservoir north of Posušje (built on a small tributary to the Ričina) in Bosnia and Herzegovina. However, it may be not native there: in 2008, local fishermen claimed that it had been introduced to the Ričina and the Tribistovo Reservoir from Imotski. At present, it is extremely rare in the entire Imotski area (based on the local population surveys). We failed to collect it in both Imotsko Polje and the Ričina River in 2017–2019.

There are also no recent records of any findings of a small-scaled *Squalius* downstream the Vrljika–Matica at present days, but *S. microlepis* phenotype 1 inhabits a small karstic lake, Krenica, which is located in the south of the Drinovci hill and is fed by underground waters of the Vrljika-Matica. So, it appears that Krenica Lake, populated by *S. microlepis* phenotype 1 and the lower reaches of the Vrljika-Matica, populated by phenotype 2, are the closest known localities of the ranges of the two phenotypes.

Some indications in literature allow to assume that *S. microlepis* of the Imotski area is a lacustrine species rather than a riverine one. Karaman (1928: 159–160) indicated that *S. microlepis microlepis* prefers ‘calm’ water and was found in Prološko Lake but not in the Vrljika River stream. All individuals ever observed by the authors of this study in the Imotski area were from Prološko Blato. Outside Imotski, Karaman (1928: 159) mentioned that Kolombatović (without an exact reference) had found this fish in Baćina lakes in lower reaches of the Neretva, in stagnant waters only, and never in the Neretva stream. *Squalius microlepis* (as *Leuciscus turskyi microlepis*) was considered as a lacustrine species by Vuković and Ivanović (1971: 150–151).

Matica-Tihaljina-Trebižat karst system

All specimens examined except two found in the karst river system of Matica-Tihaljina-Trebižat of the Neretva drainage belong to *S. microlepis* phenotype 2. The most upstream locality is the lower reaches of the Vrljika-Matica and the Grude Canal at its confluence with the Matica at Drinovci; this locality is close to the terminus of the river. The Vrljika-Matica originates in the northwest of Imotsko-Bekijsko Polje in Croatia. In natural conditions, the river used to go underground in a ponor (swallow hole, or sinkhole) south of the Drinovci hill, now it is accumulated in a lake, and water passes through a tunnel to the Tihaljina River some 150 m below, where a small electric power plant is constructed. The Tihaljina comes from underground very close to this point at the foot of the Jagodnica Mountain south of Drinovci as a strong karst spring, which is a continuation of the Matica underground stream (Bonacci et al. 2013b). It goes southeast to Klobuk Mountain and the spring Klokun, where it changes its name to Mlade, and from Humac to the confluence with Neretva it is called Trebižat. Tihaljina-Mlade-Trebižat is 50 km long. We are not aware of any collection samples of a small-scaled *Squalius* from this river section that we could additionally examine. As *S. microlepis* phenotype 2 is only recorded downstream to Grabovnik-Vašarovići, we suppose that it does not occur below the Kravica Waterfall (ca. 43°9'22"N, 17°36'29"E); this should be checked indeed.

**Figure 10.**
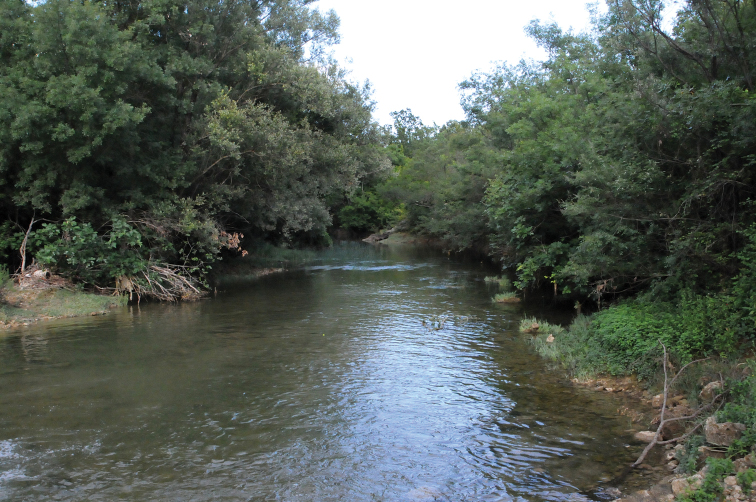
A locality where two phenotypes of *Squalius microlepis* co-occur: Tihaljina River at Tihaljina, Bosnia and Herzegovina (7 July 2011).

Two specimens (J and K) from the Tihaljina River in the village of Tihaljina (Fig. 10) were unambiguously identified as *S. microlepis* s. str. (phenotype 1) using the diagnosis presented above and clear assigned to this phenotype in statistical analyses (Table 6, Fig. 9C). Our hypothesis is that individuals of phenotype 1 could penetrate from the Imotsko Polje-Vrljika system down to the Tihaljina via existing underground karst flows though the isolation between the two was enough to support the two morphologically distinct groups of populations. A similar phenomenon of migration was discovered in this karst system for sympatric *Delminichthys adspersus* (Palandačić et al. 2012).

According to local fishermen information, after a severe drought some ten years ago, *S. microlepis* has not been found in the Tihaljina near the village of Tihaljina, and *S. tenellus* was introduced to the Tihaljina from Buško Lake but did not establish (N. Ančić, pers. comm. 2011–2019).

Poljes at Vrgorac and Gradac

Historical NMW material includes specimens from at Gradac and Vrgorac, some indicating karst poljes’ names (Jezero and Dusina). Polje Jezero is a wetland (blato) with a periodical lake and the sinking stream Matica [Vrgoračka Matica, not to be confused with Vrijeka-Matica in Imotsko Polje] as a part of the right-hand tributary system of the Neretva. The Dusina area, where some karstic streams form temporary lakes, is located near Polje Jezero and belongs to the same karst drainage system. *Squalius microlepis* was often reported from Polje Jezero and ‘Lake of Dusina’ in the past since its original description based on NMW specimens (e.g., Heckel and Kner 1858: 206, Canestrini 1865: 67, Canestrini 1866: 111, Kolombatović 1886: 16, Car 1911: 64). Mrakovčić et al. (1996) indicate the occurrence of *Squalius microlepis* in the lower part of the Matica in Polje Jezero; however, only one specimen was collected by him many years ago (M. Mrakovčić, pers. comm.).

There were only five specimens from this area available for examination added by two more specimens we supposedly attribute to it. This sample is quite morphologically heterogeneous. Specimens C and E unambiguously belong to *S. microlepis* s. str. (phenotype 1) and F and G (Fig. 6A) to phenotype 2 (Table 6, Fig. 9C). Specimens A (Fig. 6C), B and D (Fig. 6B) are intermediates between the two phenotypes.

Specimen B (NMW 49427) is labelled as ‘Narenta, Heckel Reise 1840’ but there is no clarifying information on the exact locality. We failed to find collection specimens or reliable records on *S. microlepis* from the Neretva main stream (a long list of publications checked by us can be requested from the corresponding author). We speculate that “Neretva” as a locality can refer to streams near Vrgorac or the drainage in general; for example, Seeley (1886: 169–170) and Car (1911: 64) mentioned “river Neretva near Vergorac”. Karaman (1928) indicated that he had never found *S. microlepis* in the Neretva main stream.

We hypothesise that both phenotypes could co-occur in the poljes near Vrgorac in the past or individuals of the phenotype 2 from the upstream karstic system of the Tihaljina could migrate downstream to the poljes at Vrgorac. They could probably hybridise as some specimens are of intermediate morphology. The Matica [not to be confused with Vrijeka-Matica in Imostko Polje] is a part of the right-hand tributary system of the Neretva and connected to the Tihaljina system in its northernmost (upper) part (Bonacci et al. 2013b). We failed to find this fish during intensive field trips in karst poljes near Vrgorac in 2017–2019.

Two specimens NMW 49228 (as 49227 in Bănărescu and Herzig-Straschil (1998: 419)) labelled ‘Zara’ (an Italian name for Zadar, a town on the Croatian coast, ca. 44°7'19"N, 15°16'20"E), are also identified by our analyses as *S. microlepis* phenotype 1. Bănărescu and Herzig-Straschil (1998) supposed that these two specimens do not belong to this species but did not offer an alternative hypothesis. However, no other specimens of a small-scaled *Squalius* are known from this area considerably remote from the main range of distribution of *S. microlepis* s. l. In the vicinities of Zadar, there was a lake, Bokanjačko Jezero, dried up long ago. At present, the Baštica River and two artificial lakes in that region are inhabited by *Rutilus aula* (Bonaparte) and a wide range of introduced species (e.g., *Rutilus rutilus* (L.), *Squalius cephalus* (L.), *Lepomis gibossus* (L.), *Carassius gibelio* (Bloch), *Cyprinus carpio* L., *Ameiurus melas* (Rafinesque) (unpublished data of DJ and PZ). Most probably, the label does not refer to Zadar, but the sample might have been sent to NMW from Zadar by Kolombatović. The NMW collection contains two more samples labelled as “Kolombatović, Zara” (no date), of *Chondrostoma knerii* (Heckel, 1843) and *Scardinius plotizza* (Heckel & Kner, 1858) that indicates the Neretva drainage.

### The Cetina River *Squalius*

The Cetina River is also sometimes included into the range of *S. tenellus* (Freyhof and Kottelat 2008; Ćaleta et al. 2015), but it is not clear, if the species is considered as introduced or native. We could not find a published morphological description of the Cetina fish that supports this opinion. On the contrary, the native Cetina *Squalius* was identified as *S. microlepis* by some earlier authors (Kolombatović [Kolombatowitch] 1886, Brusina 1907). Later, it was considered a new undescribed species (Zupančič 2007: *Squalius* sp. 4) but a formal description did not follow. In the most recent review of Croatian freshwater fishes (Ćaleta et al. 2019) the presence of *S. microlepis* in the Cetina is considered as not confirmed.

**Figure 11.**
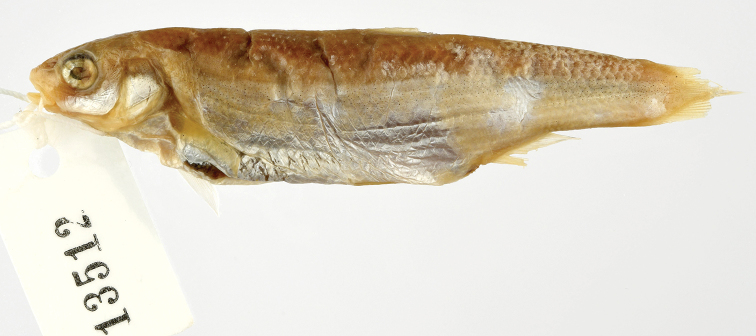
Specimen MZUF 13512 (identified as *S. microlepis* by Kolombatović), Cetina River. Photo credit: Saulo Bambi, Sistema Museale dell’Università degli Studi di Firenze, Sez. di Zoologia “La Specola”, Italy.

A historical specimen, collected by Kolombatović in the Cetina (MZUF No. 13512, donated from Kolombatović, June 1880; see Nocita and Vanni 1999: 214) was only examined by us from a photo (Fig. 11). The specimen is damaged, the number of scales in the lateral series can be calculated by the scale pockets and remaining scales, and it is 69. So, it cannot be identified as *S. tenellus* but is similar to *S. microlepis* by this count though being quite different from the latter by its general appearance and may be *Telestes ukliva* (Heckel, 1843) which is a species endemic to the Cetina.

### Taxonomy vs. variations and variability

The three small-scaled entities, *S. tenellus*, *S. microlepis* s. str. (phenotype 1) and *S. microlepis* phenotype 2, appear much better morphologically differentiated from each other than species within the *S. cephalus* group (see, e.g., Doadrio et al. 2007; Turan et al. 2009; Bogutskaya and Zupančič 2010; Özuluğ and Freyhof 2011). Four published cytb sequences of *S. microlepis*, two from the Krenica Lake and two from the Trebižat River (Freyhof et al. 2005; Perea et al. 2010; Schönhuth et al. 2018), show some genetic difference between the two localities, 0.53–0.67% (R. Šanda, pers. comm.). We did not have the possibility to examine the voucher specimens, but the Krenica specimens are most probably a true *S. microlepis* (phenotype 1) and the Trebižat specimens might represent a *S. microlepis* phenotype 2. No variability was found between five published sequences of CO1 (Perea et al. 2010, Geiger et al. 2014, Schönhuth et al. 2018) – three from the Krenica-Imotski area and two from the Tihaljina-Trebižat (R. Šanda, pers. comm.).

Readily morphologically diagnosable entities cannot always be taxonomically discriminated using molecular markers due to very rapid events of speciation (i.e., species radiations) and specific factors driving them, such as niche evolution or morphological key innovations (e.g., Bickford et al. 2007; Martin et al. 2016) forming species complexes or polymorphic species. For example, the CO1 marker did not provide resolution in at least 17 complexes of “closely related” conventional (clearly morphologically distinct) species in the subfamily Leuciscinae (Geiger et al. 2014: table S1-C). On the other hand, many intraspecific morphological differences can occur and express themselves, for example, as ecological variability or geographic variation. Polymorphic populations are more the rule than the exception in fish (Skulason and Smith 1995) as differences between habitats of fishes (e.g., related to flow regime or foraging opportunities) create selective pressures resulting in morphological divergence between conspecific populations (Langerhans et al. 2003; Senay et al. 2014).

The key issue is how to interpret the morphological differentiation in these groups – either as reflecting different nominal species or as representing varieties or (eco-) phenotypes within a single species. As very limited molecular data exist on the two phenotypes of *S. microlepis*, we refrain from any taxonomic and nomenclatural conclusions until new molecular approaches (and new markers) are used, the polymorphism is properly sampled, and much more specimens are available for genetic phylogenetic analyses. However, as shown above, we can hypothesise that the phenotype 1 might represent a lacustrine morph of the species while the phenotype 2 is a riverine one.

### Conservation implications

Our study emphasises the fact that *S. microlepis*, either a group of two putative species or two habitat-related phenotypes, has become extirpated or extremely rare in the most part of its range since 2004–2011. A reason of the dramatic decline may be due to introductions of *Perca fluviatilis* Linnaeus, *Squalius cephalus* Linnaeus and *Esox lucius* Linnaeus established throughout the region. Hence, the phenotypic diversity described in the paper has been already largely lost and a critical investigation of its conservation status is severely required based on population genetic data. We applied the IUCN criteria (3.1) and suppose that the Red List status of the species should be Critically Endangered (CR: A2ce) based on 90% population reduction estimated in the last 15 years (ca. three generations). Sub-criteria: (c) population size reduction observed through the decline in the area of occupancy (AOO) and the extent of occurrence (EOO), and (e) effects of introduced taxa, pollutants and competitors are in place. Exact causes of the reduction are not yet known and may have not ceased. Remaining EOO has been estimated as approximately 250 km^2^ and AOO only around 20 km^2^ (five 2 x 2 km cells), although the lack of data since 2011 makes the situation even more critical.
